# The ribonuclease RNase T2 mediates selective autophagy of ribosomes induced by starvation in *Saccharomyces cerevisiae*

**DOI:** 10.1016/j.jbc.2025.108554

**Published:** 2025-04-26

**Authors:** Atsushi Minami, Kohei Nishi, Rikusui Yamada, Gai Jinnai, Hikari Shima, Sakiko Oishi, Hirofumi Akagawa, Toshihiro Aono, Makoto Hidaka, Haruhiko Masaki, Tomohisa Kuzuyama, Yoichi Noda, Tetsuhiro Ogawa

**Affiliations:** 1 Department of Biotechnology, The University of Tokyo, Bunkyo-ku, Tokyo, Japan; 2 Agro-Biotechnology Research Center (AgTECH), The University of Tokyo, Bunkyo-ku, Tokyo, Japan; 3 Collaborative Research Institute for Innovative Microbiology (CRIIM), The University of Tokyo, Bunkyo-ku, Tokyo, Japan

**Keywords:** autophagy, molecular evolution, ribonuclease, ribosome, structural model

## Abstract

RNase T2 is a conserved ribonuclease, playing essential and diverse roles despite its simple enzymatic activity. *Saccharomyces cerevisiae* RNase T2, known as Rny1p, is stress-responsive and localizes in the vacuole. Upon starvation, ribosomes are degraded by autophagy, in which Rny1p mediates rRNA degradation. However, whether the ribosomal degradation is selective or nonselective is still being determined in *S. cerevisiae*. Here, we elucidated novel aspects of ribosome degradation mechanisms and the function of Rny1p in stress response. We discovered that most ribosomes are selectively degraded, whose mechanism differs from the previously reported selective degradation process called “ribophagy.” Rsa1p, a factor involved in assembling 60S ribosomal subunits, is revealed to interact with Atg8p and act as a receptor for selective ribosome degradation in the cytosol. The accumulation of rRNA in vacuoles, due to lack of Rny1p, leads to a decrease in nonselective autophagic activity. This is one of the reasons for the inability of Rny1p-deficient strains to adapt to starvation conditions. Rny1p is also reported to be secreted and associated with the cell wall. We revealed that a C-terminal extension of Rny1p, characteristic in some fungal RNase T2, is required to anchor the cell wall. Some nonfungal RNase T2 proteins also have C-terminal extensions. However, their sequences and structures differ from those of fungal RNase T2, suggesting that their biological functions may also be distinct. The diversity of C-terminal extensions across different organisms is thought to be one reason why RNase T2 plays various roles.

Cells contain numerous proteins, and ribosomes serve as platforms for synthesizing these proteins. About 2000 ribosomes were estimated to be produced per minute in each cell during the exponential growth phase. Remarkably, the volume occupied by these ribosomes constituted up to 40% of the total cell volume (1). Because protein synthesis consumes enormous amounts of energy, the cell constantly monitors and controls the activity of ribosomes to balance the energy status. When nutrients are abundant, the cells actively synthesize ribosomes. Conversely, ribosome biogenesis and initiation of protein synthesis are promptly inhibited when the nutrient source is depleted. These responses prevent energy wastage in nutritionally restricted environments.

Ribosomes have been reported to be degraded in response to rapamycin treatment in *Saccharomyces cerevisiae* (2). Rapamycin induces a nutrient starvation response in cells by inhibiting target of rapamycin complex 1 (TORC1), crucial for nutrient source sensing. This result indicates that upon nutrient starvation, intracellular ribosomes undergo degradation in addition to suppressing new ribosome synthesis and translation initiation. However, details of this degradation mechanism remain unclear.

We studied the molecular function of the conserved ribonuclease RNase T2. RNase T2 is present in almost all organisms with sequenced genomes and in some viruses. RNase T2 is a lysosome/vacuole-localized acidic enzyme with nonspecific cleavage activity against ssRNA (3). Notably, it has been demonstrated that most RNase T2 degrade rRNA (4, 5, 6, 7). Although the enzymatic function of RNase T2 is simple, it plays diverse and essential roles in organisms. For example, in humans, RNase T2 has been suggested to function as a cancer suppressor (8). Deletions and mutations in the *RNASET2* gene have been reported in patients with cystic leukoencephalopathy (9). RNase T2 has been implicated in innate immunity (10, 11, 12, 13). In plants, RNase T2, also known as S-RNase, is involved in self-incompatibility and discriminates between self and nonself cells in Solanaceae, Rosaceae, and Plantaginaceae (14, 15). In viruses, RNase T2 attacks the immune cells of the infected hosts (16, 17, 18). RNase T2 is also present in the budding yeast and is called Rny1p. Rny1p localizes in the vacuole, and possesses the N-terminal signal peptide, four glycosylation sites, and the C-terminal extension region whose functions remain unknown. (19, 20). RNase T2 also contains two conserved motifs called catalytic active sites (CASI and CASII), and two histidine residues, His87 and His160, are included in each motif (21) (Fig. 1*A*). This RNase is stress-responsive; for example, Rny1p is required for growth at high temperatures and under high osmotic pressures (19).

**Figure 1:**
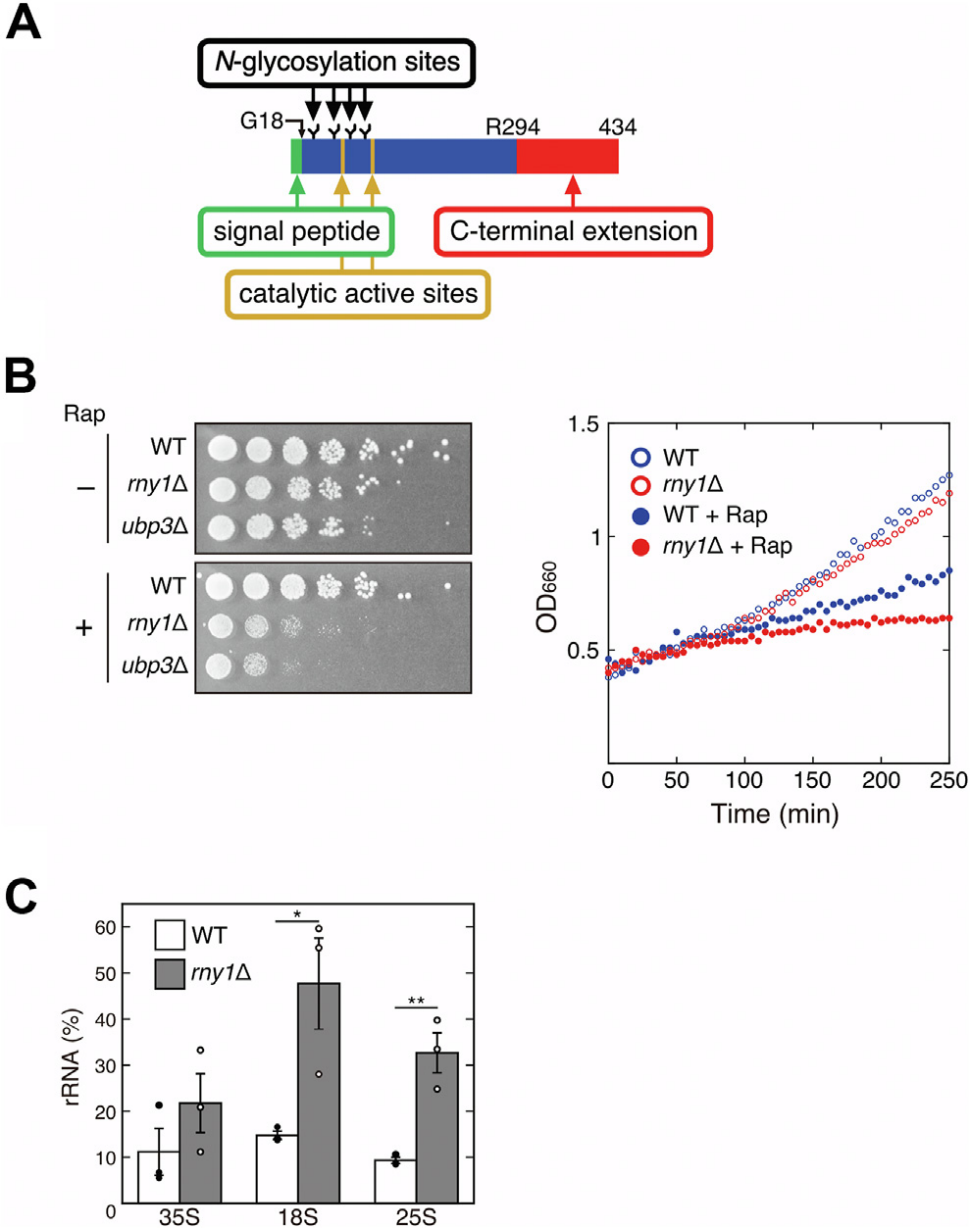
**Rapamycin induces Rny1p-dependent rRNA degradation.***A*, the schematic structure of Rny1p (434 amino acids) is shown. The signal peptide, composed of 18 amino acids, is indicated. Catalytic active sites (CASI and CASII) contain catalytic residues His87 and His160, respectively. R294 represents Arg294, corresponding to the N-terminus of the C-terminal extension. Rny1p contains four *N*-linked glycosylation sites (N37, N70, N103, and N123) (20). *B*, the sensitivity of WT and *rny1*Δ strains to rapamycin was evaluated by spot test using YPD solid medium containing 5 nM of rapamycin (*left* panel). The *rny1*Δ strain is sensitive to rapamycin as well as the *ubp3*Δ strain. The effect of rapamycin on the cell growth of WT and *rny1*Δ strains was also examined using YPD liquid medium containing 100 nM of rapamycin (*right* panel). The doubling times for the WT strain, *rny1*Δ strain, and the addition of rapamycin to each, calculated from the graph on the right, were 143, 192, 275, and 699 min, respectively. The cell growth of *rny1*Δ was severely suppressed by the rapamycin treatment. *C*, one hundred nanomolars of rapamycin was added to the WT and *rny1*Δ strains in the mid-log phase. After 3 h of rapamycin treatment, quantitative RT-PCR (RT-qPCR) analysis was performed. The amount of rRNA remained after rapamycin treatment was calculated as follows: rRNA (%) = 100 × (amount of RNA prepared from cells with rapamycin treatment for 3 h)/(amount of RNA prepared from cells without rapamycin treatment for 3 h). Data are presented as the means ± standard error of three independent experiments. Asterisks indicate significant differences (∗*p* < 0.05, ∗∗*p* < 0.01, Student’s *t* test). 18S and 25S rRNAs were abundant in the *rny1*Δ strain compared to the WT strain after the addition of rapamycin.

By analyzing the function of Rny1p, we obtained novel insights into ribosomal degradation in response to autophagy-mediated nutrient starvation. Autophagy is a conserved mechanism in which a bilayer membrane, known as an autophagosome, envelops a targeted object. After the fusion of the outer membrane of the autophagosome with the vacuolar membrane, the inner membrane of the autophagosome, termed the autophagic body, is quickly degraded by proteases and releases its cytosolic contents in the vacuole. Two types of autophagy are known: nonselective (bulk) and selective. The former randomly degrades cytoplasmic components, whereas the latter selectively recognizes and degrades specific factors. However, the pathway utilized for ribosomal degradation in yeast remains to be established. In this study, we indicate that ribosomal degradation depends mainly on selective autophagy, which differs from the previously reported mechanism known as “ribophagy” (22). Selective autophagy requires receptors to specify factors for degradation, and we discovered that Rsa1p, a nuclear factor involved in the assembly of 60S ribosomal subunits, functions as a ribosome-specific receptor. Furthermore, we propose a molecular mechanism for ribosomal degradation based on structural prediction data and biochemical experiments. In addition to the localization in the vacuole, Rny1p has also been reported to be secreted outside the cell and interact with the cell wall (20). We have shown that the C-terminal extension, characteristic of Rny1p, is required for this binding. We also suggested that the extension domain binds to the cell wall using the sugar chains of the cell wall as a scaffold. This C-terminal region may be one of the reasons for the diversity of RNase T2 roles in species.

## Results

### Rapamycin treatment–induced rRNA degradation depends on a different pathway than ribophagy

First, the sensitivity of the *rny1*Δ strain to rapamycin, a TOR inhibitor, was examined by spot test using a Ubp3p-deficient strain, which is reported to be sensitive to rapamycin, as a control (22). The colony formation of *rny1*Δ cells was severely reduced on the rapamycin-containing plate as well as the *ubp3*Δ strain compared to the WT strain (Fig. 1*B*, left). We also examined the effect of rapamycin on the growth of WT and *rny1*Δ strains in liquid culture. Rapamycin reduced the growth rate of the WT strain, but it continued to grow. In contrast, the growth rate is dramatically reduced in the *rny1*Δ strain (Fig. 1*B*, right; the doubling times of each strain were indicated in the legend). These results suggest a link between Rny1p and the starvation response to rapamycin.

Next, to examine whether rapamycin-induced rRNA degradation depends on Rny1p, we extracted total RNA from the WT and *rny1*Δ strains with or without rapamycin treatment and performed electrophoresis using denaturing agarose gel. Degradation of 18S and 25S rRNA was suppressed in the *rny1*Δ strain compared to the WT strain. On the other hand, no significant difference was observed for 35S rRNA (Fig. S1*A*). The extent of degradation was quantified in more detail by RT-qPCR. Consistent with the result obtained from electrophoresis, 18S and 25S rRNA prepared from rapamycin-treated *rny1*Δ cells were more abundant than those from rapamycin-treated WT cells. No difference in 35S rRNA was observed between the WT and the *rny1*Δ strains, which supports the result shown in Figure S1*A* (Fig. 1*C*). We further performed an *in vivo* nucleic acid staining of rapamycin-treated WT and *rny1*Δ strains (23). Nucleic acid–derived fluorescence was observed in the vacuoles of the *rny1*Δ cells but not in the WT cells after rapamycin treatment (Fig. S1*B*). These results indicate that Rny1p is involved in rapamycin-induced degradation of rRNAs within mature ribosomes.

**Figure 2:**
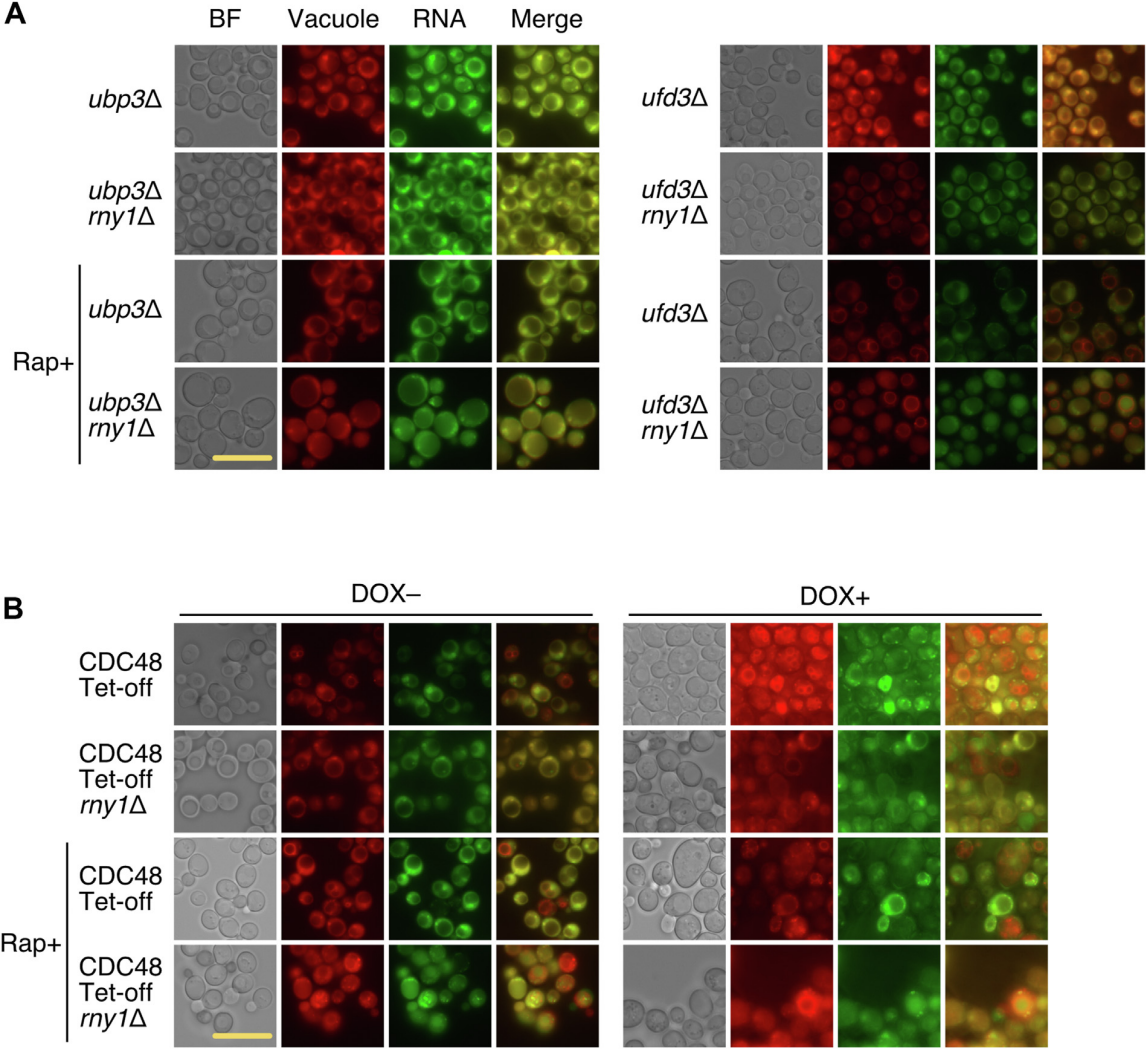
**The selective autophagy of ribosomes shown in this study differs from that of previously reported ribophagy.***A*, the strains, as indicated, were grown to the mid-log phase with or without rapamycin (100 nM) for 2 h. The vacuoles and RNA were stained, and fluorescence microscopic analysis was performed. RNA accumulation was not observed in the vacuole of strains lacking Ubp3p or Ufd3p, whose factors are reported to be required for ribophagy, after the rapamycin treatment. *B*, the CDC48 Tet-Off strain, which expresses Cdc48p in the absence of doxycycline (DOX), and that lacking Rny1p were cultivated in a YPD medium without DOX; Cdc48p is also reported to be involved in ribophagy. After growing these strains to the mid-log phase, rapamycin was added, and these strains were further cultivated for 2 h, followed by cell staining. At the same time, these strains were grown in a DOX-containing YPD medium and cultivated for 14 h to suppress the expression of the *CDC48* gene. Rapamycin was added, and the cultivation continued for a further 2 h. Then, the cells were sampled, RNA and vacuoles were stained, and fluorescence microscopic analysis was performed as in (*A*). RNA accumulation was not observed even in the vacuole of the Cdc48p-depleted strain. The scale bars in (*A*) and (*B*) represent 10 μm.

Previously, ribosomes have also been shown to be degraded by a selective autophagy process known as “ribophagy” in *S. cerevisiae* upon nitrogen starvation (22). It was reported that ribophagy requires Ubp3p, Ufd3p, and Cdc48p (22, 24); Ubp3p is a ubiquitin protease (22), while Ufd3p-Cdc48p is required for ubiquitin-mediated proteolysis (25). If the rRNA degradation induced by rapamycin depends on known ribophagy, the lack of these factors will cause undegraded rRNA to accumulate in the vacuole. Therefore, we performed nucleic acid staining of the rapamycin-treated each strain shown in Figure 2 (23). In the strains depleted of Ubp3p or Ufd3p, RNA was not observed in the vacuoles, while in those additionally lacking Rny1p, RNA accumulation in the vacuoles was observed, showing that these factors are all dispensable in rapamycin-induced RNA degradation in the vacuole (Fig. 2*A*). *CDC48* cannot be disrupted as it is an essential gene. Therefore, we used a strain that expresses Cdc48p only in the absence of doxycycline (DOX, CDC48 Tet-Off). After suppressing the *CDC48* expression by culturing in a DOX-containing medium, we treated the cells with rapamycin for 2 h. However, even in this case, no accumulation of RNA in the vacuole was observed, and RNA accumulation was observed in the strain in which the *RNY1* gene of CDC48 Tet-Off strain was disrupted (Fig. 2*B*). Based on the above results, Ubp3p, Ufd3p, and Cdc48p are not involved in RNA degradation in the vacuole, indicating that the RNA degradation shown here depends on a pathway different from known ribophagy.

While our experiments were in progress, Huang *et al.* reported in detail on the metabolome of RNA degradation caused by nitrogen starvation, particularly from the perspective of nonselective autophagy. They also indicated that Rny1p is required for the first step of the degradation process (23). Furthermore, they showed that Ubp3p and Bre5p, a cofactor of Ubp3p, had little effect on nitrogen starvation (23); our results are consistent with this. Similar to rapamycin, nitrogen starvation also inhibits TORC1 activity. Therefore, experiments were hereafter conducted under nitrogen-starved conditions instead of rapamycin treatment.

### Rsa1p is involved in selective ribosome degradation

Huang *et al.* reported that a certain number of ribosomes are nonselectively delivered to the vacuole by autophagy under nitrogen starvation (23). In other words, the existence of selectively degraded ribosomes is yet to be completely ruled out. Hence, the existence of a pathway other than ribophagy is suggested. Recently, it was reported that when mTORC1, a homolog of yeast TORC1, is inactivated, a protein called nuclear fragile X mental retardation–interacting protein 1 (NUFIP1), that is normally present in the nucleus, interacts with and delivers ribosomes to autophagosomes for selective degradation in mammalian cells (26). We assumed that yeast ribosomes are also degraded by selective autophagy and that Rsa1p, a functional homolog of NUFIP1 in *S. cerevisiae*, is involved in this process. Rsa1p localizes to the nucleus and serves as a platform for box C/D small nucleolar ribonucleoprotein assembly (27, 28, 29) as well as NUFIP1.

**Figure 3:**
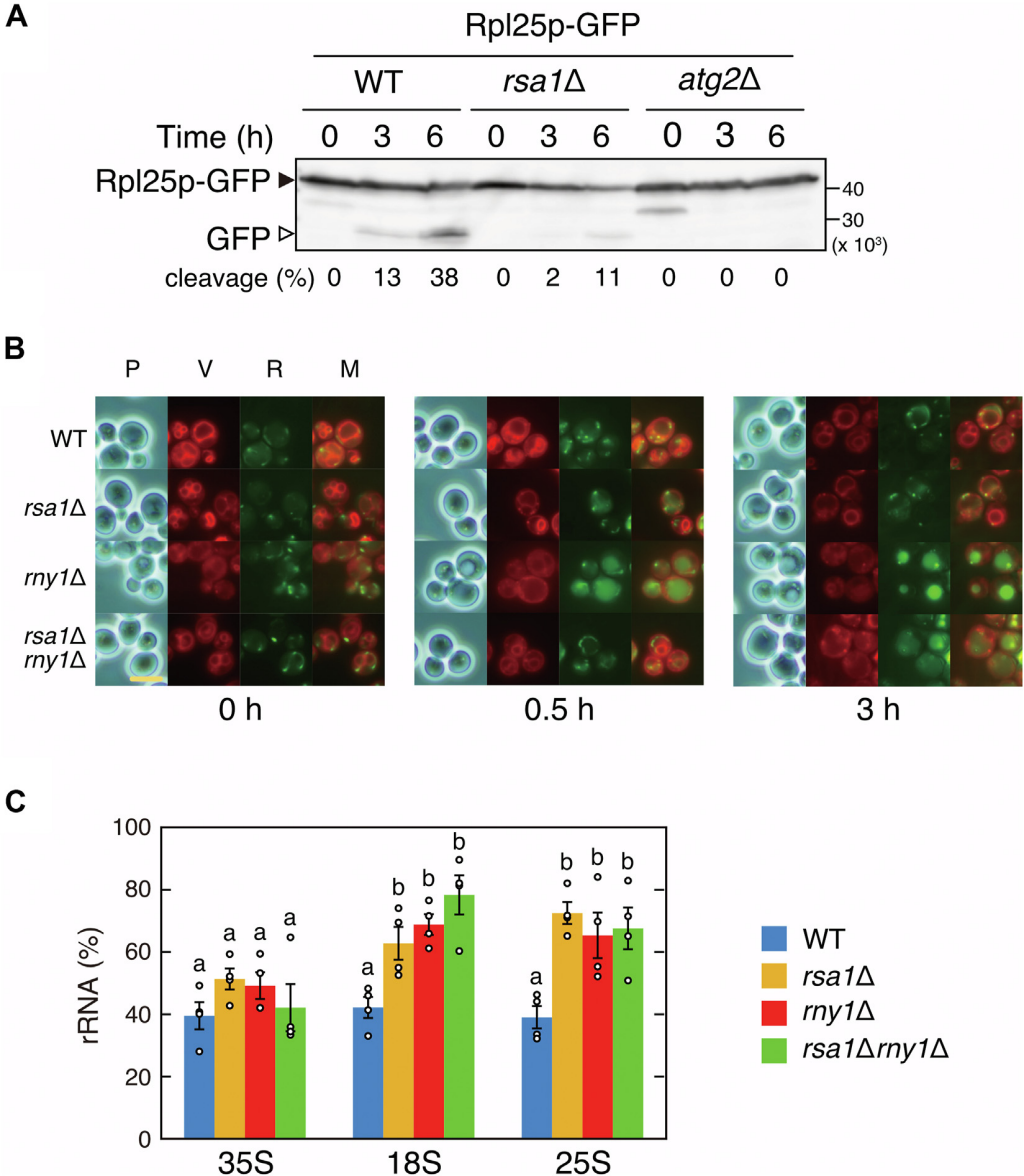
**Most ribosomes are degraded in an Rsa1p-dependent manner under nitrogen starvation.***A*, WT, *rsa1*Δ, and *atg2*Δ strains chromosomally expressing the GFP-tagged ribosomal protein (Rpl25p-GFP) were cultured in nitrogen-depleted SD medium (SD (-N) medium). An equal volume of cell culture was collected at the indicated time points, and then cell lysates were prepared. Western blotting was followed using an anti-GFP antibody. Note that the total amount of protein is not standardized, so comparing the amount of proteins in different lanes cannot be evaluated. This experiment was conducted twice, and both obtained the same results. One of these is shown as representative of the other. The percentage of the GFP cleavage, which indicates the progress of autophagy, was calculated as follows: cleavage (%) = 100 × (intensity of band from GFP fragment)/(intensity of band from Rpl25p-GFP + intensity of band from GFP fragment). The proportion of GFP fragments to Rpl25p-GFP was lower in the *rsa1*Δ strain than in the WT strain. *B*, WT, *rsa1*Δ, *rny1*Δ, and *rsa1*Δ*rny1*Δ strains were grown in SD (-N) medium, and vacuoles and RNA were stained at the indicated time points. “P” labeled above the panels indicates the observation was made by phase-contrast microscopy, while “V” and “R” indicate the observation of cells in which vacuoles or RNA were stained, respectively. “M” indicates a merged panel with “V” and “R.” RNA accumulation in the vacuole, which was delivered but undegraded, was observed in the *rny1*Δ strain, while it was not in the *rsa1*Δ strain. The scale bar represents 5 μm. *C*, strains shown in (*B*) were grown in SD or SD (-N) medium. After 3 h of cultivation, amounts of 35S, 25S, and 18S rRNA were quantified by RT-qPCR analysis, as shown in Figure 1*C*. The amount of rRNA remaining after induction of nitrogen starvation was calculated as follows: rRNA (%) = 100 × (amount of RNA prepared from cells cultured in SD (-N) medium)/(amount of RNA prepared from cells cultured in SD medium). Data are presented as means ± standard error from four independent experiments. Multiple comparison test was conducted for 35S, 18S, and 25S rRNA. Bars with different letters are statistically significant from one another (*p* < 0.05, Tukey-Kramer test), while bars with the same letter indicate no statistical significance. Degradation of 18S and 25S rRNA was progressed only in the WT strain.

To test the assumption, the Rsa1p dependence of ribosomal protein degradation was investigated first. Here, a GFP cleavage assay was applied. The GFP cleavage assay is used to evaluate the susceptibility of the target protein to autophagy. As autophagy progresses, the target protein fused with GFP is incorporated into the vacuole and degraded. However, as GFP is stable in vacuoles, the progress of protein degradation can be evaluated by calculating the ratio of the GFP-fusion protein and GFP fragments. Therefore, ribosomal protein L25 fused with GFP (Rpl25p-GFP) was expressed from the chromosome (30), and nitrogen starvation was induced. Cell lysate was prepared at the indicated time point and they were applied to Western blotting. Here, the strain lacking Atg2p was used as a control; Atg2p is required for autophagosome formation and autophagy never occurs in the *atg2*Δ strain. Nitrogen starvation was induced and the cleavage rate of Rpl25p-GFP was calculated (see the figure legend). As a result, the cleavage rate of Rpl25p-GFP increased in WT cells over time, and approximately 38% of Rpl25p-GFP was degraded after the induction of nitrogen starvation for 6 h. Contrarily, the fragment was barely detectable in *rsa1*Δ cells; a faint band was observed after 6 h of nitrogen starvation and about 11% was calculated to be degraded (Fig. 3*A*). The GFP fragment was not detected during the experimental period in the *atg2*Δ strain, indicating that the GFP assay worked correctly. Furthermore, most of the degradation of Rpl25p was Rsa1p-dependent. However, even in the *rsa1*Δ strain, a small amount of Rpl25p degradation was observed 6 h after nitrogen starvation as described above, so the involvement of other pathways, such as bulk autophagy, cannot be completely ruled out.

Next, we investigated the involvement of Rsa1p in the rRNA degradation. After the induction of nitrogen starvation in the WT and *rsa1*Δ strains, cells were collected at the indicated time points, and nucleic acid staining (23) was performed as shown in Figures 2 and S1*B*. In *rsa1*Δ cells, RNA accumulation in the vacuoles was not observed. This raised two possibilities: either Rsa1p did not transport ribosomes to the vacuoles or ribosomes were transported but rRNA was degraded by Rny1p. Therefore, we performed the same experiments using a strain lacking both Rsa1p and Rny1p. After nitrogen starvation for 30 min, RNA accumulation in the vacuole was not observed at all in the *rsa1*Δ*rny1*Δ strain, as in the *rsa1*Δ strain (Fig. 3*B*, middle panel). Thirty minutes of nitrogen starvation induction is sufficient for accumulating RNA in vacuoles, as RNA was remarkably observed in the *rny1*Δ strain (Fig. 3*B*, middle panel). RNA accumulation was slightly observed after 3 h of nitrogen starvation in *rsa1*Δ*rny1*Δ cells (Fig. 3*B*, most right panel). This result also suggests that other pathways, like the ribosomal protein degradation shown in Figure 3*A*, partially assist RNA degradation. To further evaluate this quantitatively, each strain shown in Figure 3*B* was cultured under nitrogen starvation conditions for 3 h. The extent of rRNA degradation was quantified by RT-qPCR, and multiple comparison test was performed on the amount of rRNA in each strain, divided into 35S, 18S, and 25S rRNA (Fig. 3*C*). A significant difference in the degradation of 18S and 25S rRNAs was observed only in the WT strain where both Rsa1p and Rny1p are present, while the degradation was suppressed in strains lacking either one of these proteins (*rsa1*Δ, *rny1*Δ, and *rsa1*Δ*rny1*Δ strains). There were no statistically significant differences in the amount of 35S rRNA between all strains. This result is consistent with that of Figure 1*C*, which indicates that the precursor rRNA is not a target in the selective ribosome degradation described here. This result confirmed that Rsa1p is required for this rRNA degradation in the vacuole. Together, the results shown in Figure 3 demonstrate that the nitrogen starvation–induced degradation of ribosomes is Rsa1p-dependent and selective.

### Rsa1p acts as a receptor for selective autophagy of ribosomes

**Figure 4:**
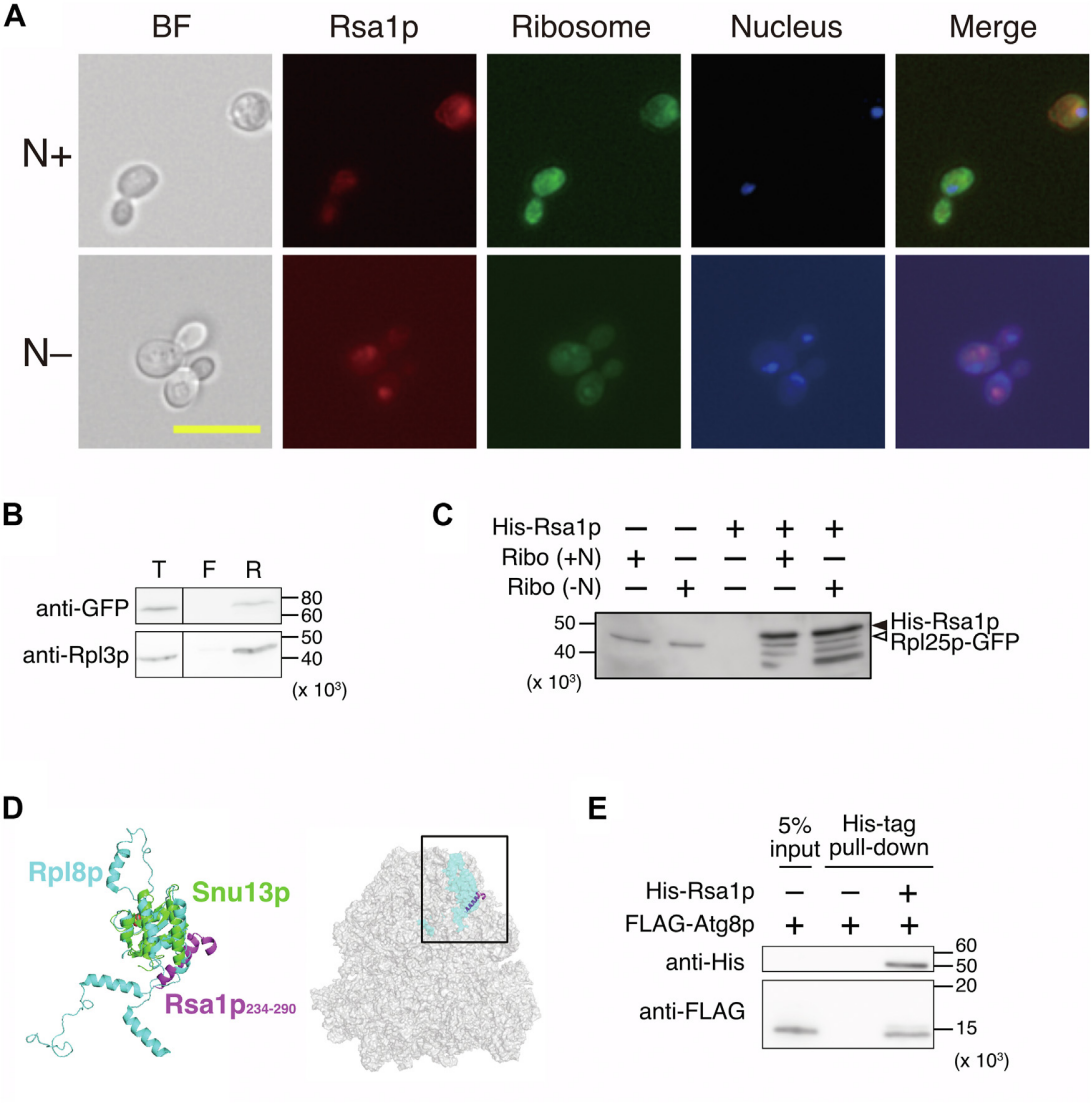
**The structural prediction and biochemical substantiates the Rsa1p-dependent selective ribosomal degradation pathway.***A*, the colocalization of ribosomes with Rsa1p was observed in yeast cells. The cells expressing mCherry-Rsa1p and Rpl25p-GFP were cultivated with or without a nitrogen source (N+ and N–, respectively). “BF” labeled above the panels indicates the observation was made by bright-field microscopy, while “Rsa1p” and “Ribosome” indicate the observation of mCherry-Rsa1p and Rpl25p-GFP in cells, respectively. “Nucleus” indicates the observation of cells in which nuclei were stained. “Merge” indicates a merged panel with “Rsa1p,” “Ribosome,” and “Nucleus.” *B*, the cellular interaction of ribosomes with Rsa1p was evaluated. Cells expressing GFP-Rsa1p were cultivated under nitrogen starvation conditions in the presence of PMSF. Cell lysate was prepared, and ribosomes were fractionated by ultrafiltration. Total cell lysate (lane “T”) and proteins in the filtrate and the retentate (lane “F” and “R,” respectively) were applied to Western blotting. *C*, the interaction of ribosomes with Rsa1p was evaluated *in vitro*. Ribosomes were prepared from cells grown with or without a nitrogen source. Proteins in the retentate from the ultrafiltration were analyzed by Western blotting. Bands corresponding to His-Rsa1p and Rpl25p-GFP were indicated as solid and open arrowheads, respectively. Extra bands were also observed in lanes containing both ribosomes and Rsa1p, which may be derived from the cleaved His-Rsa1p as Rsa1p contains the disordered regions. *D*, a model of the complex structure of Rsa1p and ribosome. In the *left* panel, the structural alignment of the ribosomal protein Rpl8p and the crystal complex structure of Snu13p-Rsa1p is shown. The Rsa1p-bound *Saccharomyces cerevisiae* 80S ribosome model is constructed based on this alignment and displayed in the *right* panel. The square in the figure indicates the assumed contact site predicted from the alignment shown in the *left* panel. *E*, the interaction of Rsa1p with Atg8p was evaluated by pull-down assay. His-Rsa1p immobilized on the magnetic beads was incubated with FLAG-Atg8p. Proteins captured in the magnetic beads were analyzed by Western blotting. Three lanes are shown: 5% of the total FLAG-Atg8p used in the assay, FLAG-Atg8p incubated on the magnetic beads without His-Rsa1p, and FLAG-Atg8p incubated with His-Rsa1p on the magnetic beads.

We demonstrated that the degradation of ribosomal protein and rRNA depends on Rsa1p (Fig. 3), suggesting that Rsa1p is involved in selective autophagy for ribosomes. Generally, selective autophagy requires a receptor to recognize and interact with the target to be degraded. We assumed that Rsa1p is the receptor for the selective autophagy of ribosomes. To examine this assumption, we tried to observe the interaction between ribosomes and Rsa1p in the cell. Rsa1p fused with a fluorescent protein mCherry at the N-terminus (mCherry-Rsa1p) was expressed from a low-copy plasmid in the strain expressing Rpl25p-GFP used in Figure 3. We expected to observe colocalization of the fluorescent foci derived from Rpl25p-GFP and mCherry-Rsa1p in the autophagosome formed in the cytoplasm; however, this did not work out due to quick degradation of ribosomes. As mentioned in the introduction, the contents to be degraded by autophagy are enclosed in an autophagic body within the vacuole, and the contents are released when proteases degrade the autophagic body’s membrane. Therefore, a protease inhibitor phenylmethylsulfonyl fluoride (PMSF) was added upon nitrogen starvation to maintain the autophagic body (31). As a result, the fluorescence spot derived from mCherry-Rsa1p overlapped well with that of Rpl25p-GFP in the autophagic body upon nitrogen starvation, while it was not when the nitrogen source was present, suggesting that Rsa1p interacts with the ribosome under the nitrogen starvation condition (Fig. 4*A*). Next, to validate the direct interaction between Rsa1p and ribosomes, cells expressing GFP-Rsa1p were cultured under nitrogen starvation conditions in the presence of PMSF as in Figure 4*A*, ribosomes were fractionated from the resulting lysate by ultrafiltration, and GFP-Rsa1p contained in the retentate and filtrate were detected by Western blotting. Indeed, GFP-Rsa1p was detected in the ribosome-containing fraction (Fig. 4*B*, lane “R”), providing direct evidence that Rsa1p interacts with ribosomes *in vivo*. Furthermore, an *in vitro* binding assay of Rsa1p and ribosomes was performed. Assuming that nitrogen starvation affects the binding between ribosomes and Rsa1p, we prepared ribosomes from yeast cells grown with or without a nitrogen source, and they were used for the *in vitro* binding assay. Consequently, we found that Rsa1p binds to ribosomes prepared from both conditions almost comparable (Fig. 4*C*). Building on this observation, we tried to elucidate the binding site of Rsa1p to the ribosome. It has been shown that NUFIP1 interacts with the 60S ribosomal subunit; however, the binding sites remain unidentified (26). Therefore, we analyzed the Rsa1p-binding site of ribosomes by structural alignment based on known structures. Previously, the crystal structure of the Rsa1p PEP domain (Rsa1p_234-290_) complexed with Snu13p was determined (PDB ID: 4NUT) (32). Snu13p is a conserved RNA-binding protein involved in mRNA splicing and rRNA maturation, and it belongs to the same superfamily as ribosomal proteins eL8A and eL8B in *S. cerevisiae*. eL8A and eL8B are paralogous proteins, with their amino acid sequences being nearly identical. In the crystal or cryo-EM structures of yeast ribosomes deposited in the Protein Data Bank (PDB), all instances of eL8 are registered as “eL8A,” so eL8A is referred to as Rpl8p to conform to the notation of other ribosomal proteins. The structure of Rpl8p in *S. cerevisiae* 80S ribosome (PDB ID: 6GQV) aligns well with that of Snu13p (2.7 Å of the RMSD) (Fig. 4*D*, left). Based on this structural alignment, we constructed a model structure of ribosome-bound Rsa1p (Fig. 4*D*, right). Rpl8p is located on the surface of the ribosome. In the predicted structure, Rsa1p bound to Rpl8p faces the solvent. Therefore, Rsa1p is suggested to bind to the 60S ribosomal subunit *via* Rpl8p.

A receptor for selective autophagy is required to bind Atg8p and facilitate the engulfment of cargo by the autophagosome. Atg8p, a ubiquitin-like protein, is essential for autophagosome membrane formation and the selective recognition of degradation substrates. In mammalian cells, NUFIP1 interacts with LC3B, a homolog of *S. cerevisiae* Atg8p, upon starvation (26). Then, an *in vitro* pull-down assay was performed using His-tagged Rsa1p and FLAG-tagged Atg8p as bait and prey, respectively. His-tagged Rsa1p was expressed and purified from *Escherichia coli* as a complex with Hit1p, according to a previous report (33), and the interaction between Rsa1p and Atg8p was detected (Fig. 4*E*). Based on these findings, Rsa1p was concluded to be a receptor for selective autophagy of ribosomes.

### Impairment of rRNA degradation decreases bulk autophagic activity

**Figure 5:**
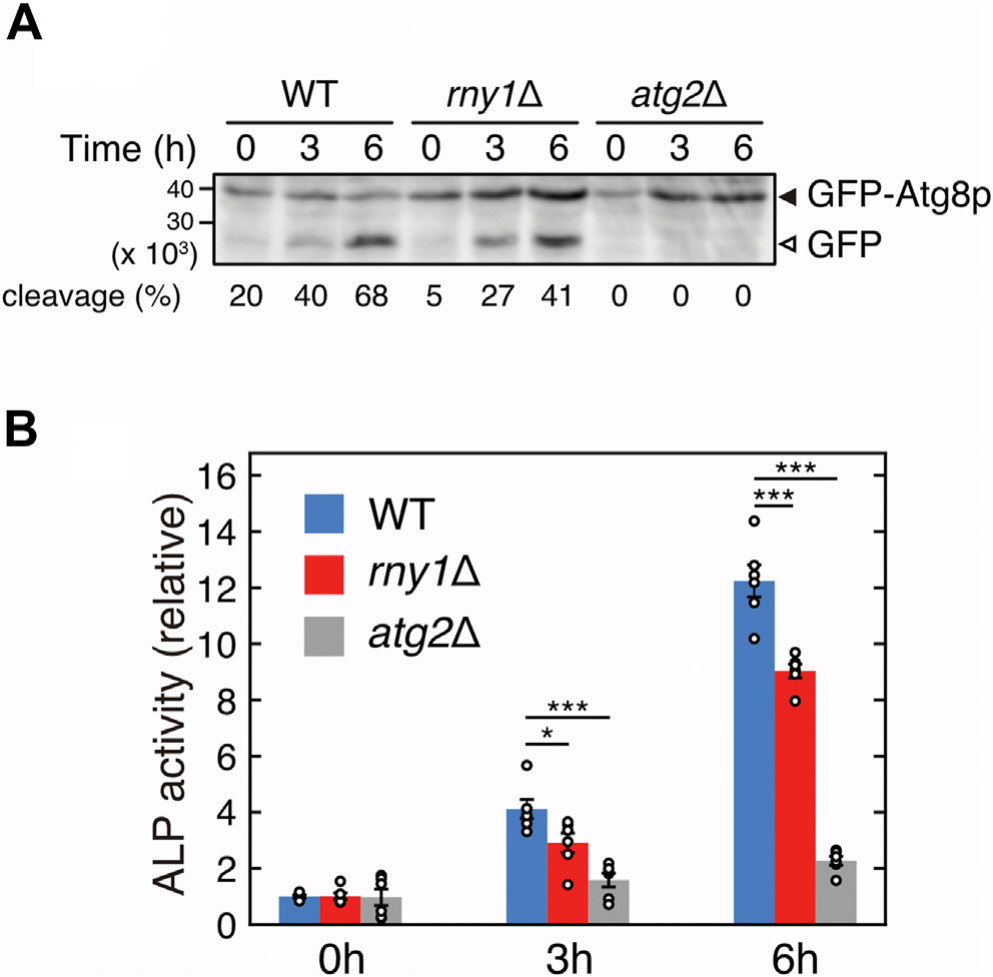
**Bulk autophagic activity is decreased in the *rny1*Δ strain.***A*, WT, *rny1*Δ, and *atg2*Δ strains expressing GFP-Atg8p were grown in SD (-N) medium and collected at the indicated time points. Cell lysates were prepared, and the GFP cleavage assay was performed. Solid and open arrowheads indicate GFP-Atg8p fusion proteins and cleaved GFP fragments. The percentage of the cleavage was calculated as follows: cleavage (%) = 100 × (intensity of band from GFP fragment)/(intensity of band from GFP-Atg8p + intensity of band from GFP fragment). The rate of GFP cleavage was lower in the *rny1*Δ strain than in the WT strain. *B*, WT, *rny1*Δ, and *atg2*Δ strains expressing the cytosolic form of Pho8p (Pho8Δ60p) were cultured in SD (-N) medium, and cell lysates were prepared at the indicated time points. Phosphatase activity was quantified to evaluate the bulk autophagic activity (ALP assay). Data are presented as the means ± standard error of six independent experiments. Asterisks indicate significant differences (∗*p* < 0.05, ∗∗∗*p* < 0.001, Student’s *t* test).

Upon starvation, Rny1p degrades rRNA; however, the degradation products of ribosomes are not recycled (23), indicating that rRNA degradation is not aimed at obtaining a nutrient source from degradation products. In *Arabidopsis*, RNase T2 (RNS2) depletion increases the number of autophagosomes, suggesting the activation of autophagy to facilitate rRNA degradation in the vacuole (34). Additionally, autophagy is partially blocked by RNase T2 deficiency in *Caenorhabditis elegans* (35). Therefore, we assumed that the cell lacking Rny1p attempts to activate autophagy; however, it failed as the rRNA occupied the vacuole, which results in the decrease of autophagic activity in *rny1*Δ cells. To test this assumption, GFP-Atg8p was expressed in WT, *rny1*Δ, and *atg2*Δ strains under a nitrogen starvation condition, and the GFP cleavage assay was performed to measure autophagic activity, as shown in Figure 3*A*. Almost 70% of GFP-Atg8p was cleaved into GFP fragments after 6 h of starvation in the WT strain. Meanwhile, approximately 60% of the GFP-Atg8p remained intact in the *rny1*Δ strain (Fig. 5*A*). To quantify the autophagic activity of WT and *rny1*Δ cells under nitrogen starvation in more detail, we performed the alkaline phosphatase (ALP) activity assay (36). The ALP assay utilizes a strain expressing Pho8Δ60p, a cytoplasmic form of the vacuole-localized ALP Pho8p that becomes activated in the vacuole. Autophagic activity can be evaluated by measuring the activity of Pho8Δ60p, which is nonselectively incorporated into the vacuole. We found that the ALP activity was lower in *rny1*Δ cells than in WT cells (Fig. 5*B*), indicating that the absence of Rny1p-mediated rRNA degradation reduces autophagic activity. Therefore, the RNA accumulation in vacuoles may be one of the reasons why the *rny1*Δ strain does not adapt to starvation.

As aforementioned, cells possess an enormous number of ribosomes. Therefore, we hypothesized that Rsa1p-mediated selective autophagy of ribosomes would compete with nonselective autophagy for autophagy-related factors. However, the GFP cleavage and ALP assays showed that the autophagic activities of WT and *rsa1*Δ strains are at similar levels after starvation induction (Fig. S2). These results indicated that Rsa1p does not perturb nonselective bulk autophagy.

### The C-terminal extension of Rny1p is not required for ribosome degradation but is necessary for cell wall anchoring

**Figure 6:**
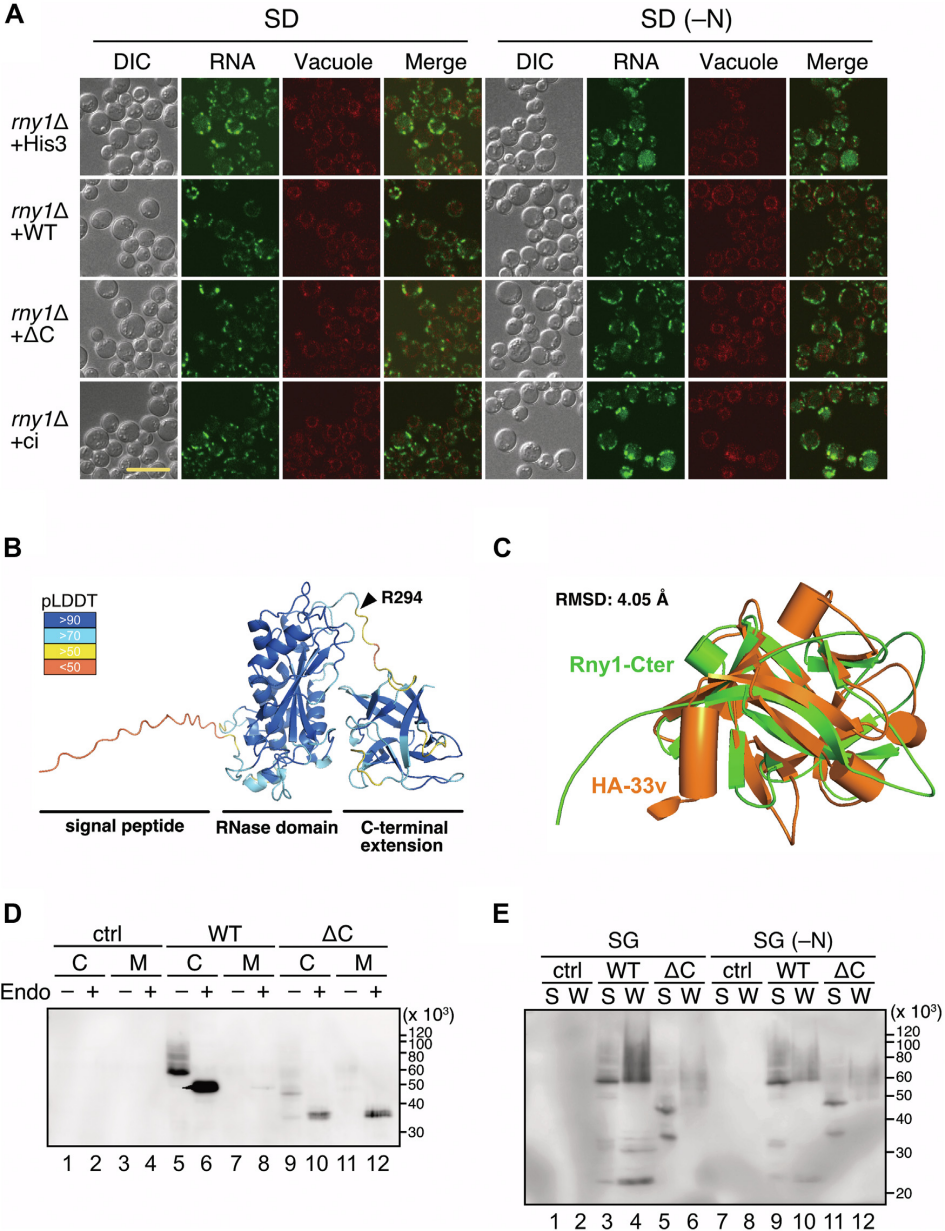
**The C-terminal extension of Rny1p is necessary to bind the cell wall.***A*, strains chromosomally expressing Rny1p-F, Rny1pΔC-F, or Rny1p-F-ci, labeled as *rny1*Δ+WT, *rny1*Δ+ΔC, and *rny1*Δ+ci, respectively, and the Rny1p-deficient strain (*rny1*Δ+His3) were cultured in SD or SD (-N) medium for 3 h. After staining the vacuoles and RNA, fluorescence confocal microscopic analysis was performed. RNA fluorescence was not observed in the vacuole of the strain expressing Rny1pΔC-F (*rny1*Δ+ΔC) as well as that expressing Rny1p-F (*rny1*Δ+WT) even in SD (-N) condition. The scale bar represents 10 μm. *B*, the structure of Rny1p was predicted using AlphaFold2 with confidence levels mapped onto the structure. The signal peptide, RNase domain, and C-terminal extension are indicated. The arrowhead indicates the 294th amino acid residue where the C-terminal extension begins, as shown in Figure 1*A*. *C*, structural alignment of the C-terminal extension of predicted Rny1p (labeled Rny1-Cter) with the crystal structure of the variable region of HA-33 (PDB ID: 5B2H, labeled HA-33v). *D*, a WT strain harboring pGMH20 (an empty vector), pGMH20-*RNY1-F*, or pGMH20-*RNY1*ΔC*-F* (leveled as ctrl, WT, or ΔC, respectively) was cultivated in an SG medium. Then, cell lysates (lanes 1, 2, 5, 6, 9, and 10) and the cultured medium concentrated 200 times by ultrafiltration (lanes 3, 4, 7, 8, 11, and 12) were prepared, and Western blotting was performed. “C” and “M” indicate cell lysate or concentrated medium loading, respectively, while “Endo” indicates endoglycosidase H treatment to remove glycosylation of Rny1p. Endo H–treated Rny1pΔC-F was observed in the medium (lane 12), while Rny1p-F was not (lane 8). *E*, strains shown in (*D*) were cultivated with (SG, lanes 1–6) or without (SG (–N), lanes 7 to 12) nitrogen source for 3 h. Then, cells were collected and treated with zymolyase to prepare spheroplasts. Spheroplasts and cell wall fraction were separated by centrifugation, and cell lysate was prepared from the spheroplast. These samples were electrophoresed, and Western blotting was performed, as shown in (*D*). “S” and “W” indicate the loading of cell lysates prepared from spheroplasts (lanes 1, 3, 5, 7, 9, and 11), or cell wall fractions (lanes 2, 4, 6, 8, 10, and 12), respectively. The cell wall–anchored Rny1p-F decreased in response to nitrogen starvation (compare lanes 4 and 10).

As reported, Rny1p is an enzyme that localizes in the vacuole (23). In addition, it has also been reported that, like some other RNase T2 proteins, Rny1p is secreted outside the cell. Furthermore, it binds to the cell wall (3, 20). Rny1p possesses the C-terminal extension (Fig. 1*A*), and it is unclear whether this region is involved in either RNA degradation or binding to the cell wall or whether it plays a completely different role. To clarify the function of this C-terminal extension, we first investigated the involvement in RNA degradation. We replaced genomic *RNY1* with *RNY1-F*, *RNY1ΔC-F*, or *RNY1*-*F*-ci (these transformants were designated as *rny1*Δ+WT, *rny1*Δ+ΔC, and *rny1*Δ+ci, respectively; see Experimental procedures) and induced nitrogen starvation in the resulting strains. Vacuolar RNA accumulation was observed in the strain lacking Rny1p (designated as *rny1*Δ+His3, see Experimental procedures) and in the *rny1*Δ+ci strain under a fluorescence microscope, while it was not observed in the *rny1*Δ+ΔC strain, as well as the *rny1*Δ+WT (Fig. 6*A*). This result indicates that the C-terminal extension is dispensable for RNA degradation in the vacuole.

Next, we analyzed the function of the C-terminal extension from a structural biology perspective because of the lack of information on homologous proteins or known domain motifs other than RNase T2 in the amino acid sequence. According to the overall structure of Rny1p predicted using AlphaFold2 (37) (Fig. 6*B*), the C-terminal extension of Rny1p is distinguishable from the structure of the RNase domain. Furthermore, we searched for protein structures similar to the predicted structure of the C-terminal extension using the Dali server (38). The top five structures (Table S4) were all known or suggested to contain domains involved in sugar binding. The predicted C-terminal extension structure was superimposed on that of the variable region of HA-33 (PDB ID: 5B2H) (39), which had the highest Z-score (Fig. 6*C*) despite the low amino acid sequence similarity between them. HA-33 is involved in the transport of a large toxin produced by *Clostridium botulinum* across the intestinal epithelial cell layer, and notably, HA-33 binds the sugar chain on the cell surface (40). The structural similarity of the C-terminal extension of Rny1p to sugar-binding domain-containing proteins, such as HA-33, suggests that Rny1p binds to the sugar chain of the cell wall *via* C-terminal extension.

We experimentally tested whether Rny1p is bound to the cell wall through its C-terminal extension. Due to the low intracellular level of the chromosomally expressed Rny1p, we prepared cell lysates from the WT strains expressing Rny1p-F or Rny1pΔC-F from the high-copy plasmid. Simultaneously, we concentrated the supernatant of the culture medium by ultrafiltration because Rny1p could not be detected unless it was enriched (Fig. S3). Western blotting showed smeared bands due to the glycosylation of Rny1p-F (labeled as WT) in the cell lysate (Fig. S3, lane 3), consistent with a previous report (20). More notably, smeared bands derived from Rny1pΔC-F (labeled as ΔC) were also detected in the cell lysate (Fig. S3, lane 5) and culture medium (Fig. S3, lane 6, extracellular Rny1pΔC-F was indicated by the asterisk). To observe Rny1p more clearly, the cell lysate and concentrated medium were treated with endoglycosidase H (Endo H) to remove the sugar chain of Rny1p. Then we found single bands of Rny1p-F, as previously reported (Fig. 6*D*, lane 6) (20). The Rny1pΔC-F was also remarkably detected in the Endo H–treated in the cell lysate and medium fractions (Fig. 6*D*, lanes 10 and 12, respectively). As mentioned above, Rny1p binds to the cell wall, but the mechanism has yet to be discovered. Our observation that Rny1p is released into the medium upon deletion of the C-terminal extension provides evidence that Rny1p interacts with the cell wall *via* the C-terminal extension. Subsequently, the differences in expression and secretion in the conditions with or without a nitrogen source were examined. We prepared spheroplasts from the strains expressing Rny1p-F or Rny1pΔC-F cultivated with or without nitrogen to separate the cytosolic (spheroplast) and cell wall fractions (referred to as “S” and “W” in Fig. 6*E*, respectively). Western blotting revealed a significant reduction of the Rny1p-F proportion in the cell wall fraction when compared to that in the cytosolic fraction upon nitrogen starvation (compare the ratio of lane 4 to lane 3 with lane 10 to lane 9). This result indicates that starvation stress alters intracellular trafficking and reduces the amount of Rny1p transferred to the cell wall.

RNase T2 plays diverse and essential roles from species to species. The length of the amino acids in the C-terminal region of RNase T2 differs depending on the host organism. In particular, RNase T2 proteins from some fungi, including Rny1p, have long extensions (Fig. S4*A*). Among them, RNase T2 in *Irpex lacteus*, referred to as Irp3, has been reported to share some critical amino acid residues in the C-terminal extension with Rny1p, suggesting that the C-terminal extensions of Irp3 may have a similar role to that of Rny1p (41). The predicted structure of the C-terminal extension of Irp3 (Fig. S4*B*) superposed well with that of Rny1p, with 2.91 Å of RMSD, which is lower than that of HA-33 (Fig. S4*C*), like those of other fungal RNase T2 proteins (Fig. S4*D*). In contrast, some RNase T2 proteins other than fungi also have C-terminal extensions, but their sequences and structures differ from those of Rny1p. For example, E^rns^, a glycoprotein produced by the classical swine fever virus, contains a motif characteristic of RNase T2 and exerts RNase activity (16, 42). E^rns^ also have a C-terminal extension required for receptor-independent translocation across the cell membrane (3). According to the prediction by AlphaFold2, the C-terminal extension of E^rns^ forms an α-helix structure (Fig. S4*E*), which is different from that of Rny1p. Other examples include the C-terminal extension of the class II plant RNase T2, which is necessary for localization to the vacuole (43) and *C. elegans* RNase T2 (35). However, their C-terminal extensions were shorter than those of Rny1p, suggesting that the structures and functions differ from Rny1p. From these analyses, it can be inferred that the diversity of C-terminal regions may have resulted from the evolution of the RNase T2 family, depending on where they function in each organism.

## Discussion

**Figure 7:**
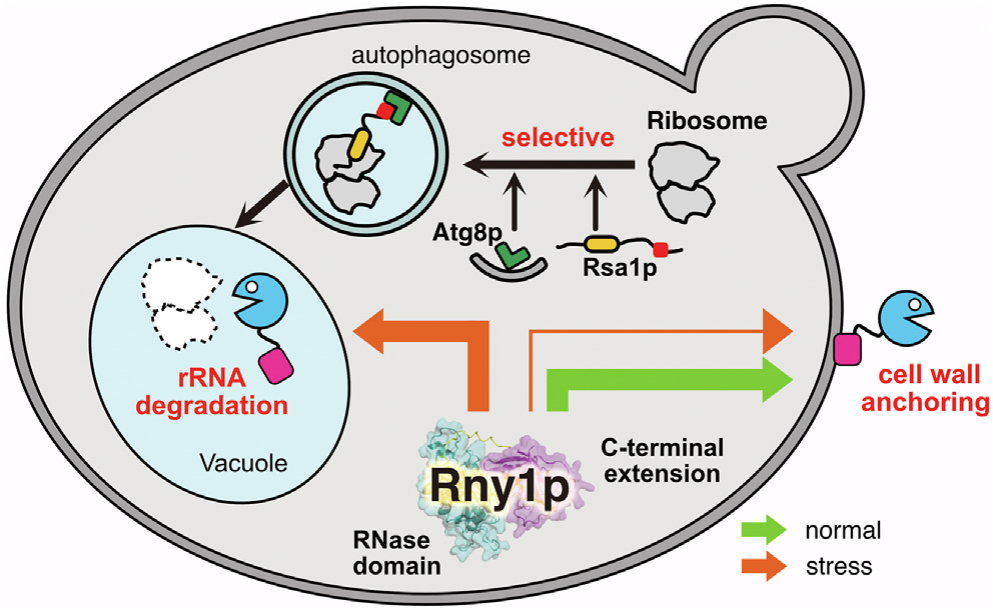
**Proposed diagram of intracellular traffic and function of Rny1p.** In *Saccharomyces cerevisiae*, Rny1p is localized in the cell wall and the vacuole. In normal conditions (*green* arrow), it is mainly secreted and binds to cell wall *via* its C-terminal extension. On the other hand, upon nutrient stress (*orange* arrow), Rny1p is localized in the vacuole to degrade ribosomal RNA. Ribosomes are transported to the vacuole in the Rsa1p-dependent manner and selectively degraded by autophagy; Rsa1p recognizes and binds to the ribosome to be degraded together with Atg8p, which is required for the autophagosome formation.

Our findings have indicated that most, if not all, ribosomes were selectively degraded in response to nitrogen starvation. This selective ribosomal degradation, mediated by Rsa1p as a receptor, differs from the process previously reported for ribophagy because factors such as Ubp3p, Ufd3p, and Cdc48p (22, 24) are unnecessary (Fig. 2). Bulk autophagy was unaffected by the massive ribosomal degradation by selective autophagy (Fig. S2). These results indicate that the factors involved in autophagy were present in sufficient amounts in the cell, showing the robustness of the autophagy pathway. The model of starvation-induced selective autophagy demonstrated by our results is shown in Figure 7. Rny1p is usually secreted from the endoplasmic reticulum *via* the Golgi apparatus and binds to the cell wall when cells are cultured with a nitrogen source. Therefore, as reported previously, Rny1p detected in the condition is in this secretory process (Fig. 6*D*, lanes 5 and 6, Fig. 6*E*, lane 3, and Fig. S3, lane 3) (20). On the other hand, Rny1p localizes in the vacuole in response to starvation (Fig. 7). Our discovery of the role of Rsa1p, a receptor for selective ribosomal degradation, shares functions with NUFIP1 in mammals. In mammals, inactivation of mTORC1 by starvation or the addition of its inhibitors results in the nuclear export of the NUFIP1 complexed with zinc finger HIT domain-containing protein 3 (ZNHIT3). Hit1p, an interacting partner of Rsa1p, is cooperatively involved in box C/D small nucleolar ribonucleoprotein assembly as ZNHIT3 does with NUFIP1 (33). Therefore, Hit1p may cooperate with Rsa1p to form a receptor for ribosomes in yeast. Ribophagy, a selective autophagy mechanism for ribosomal degradation, was first proposed in budding yeast (22, 24). In mammals, the term ribophagy is also used for selective ribosomal degradation; however, it has not been shown whether ubiquitin protease(s) corresponding to yeast Ubp3p are also required for NUFIP1-dependent ribosomal degradation (26). Our finding that Rsa1p mediates the Ubp3p-independent selective autophagy of ribosomes suggests that the NUFIP1-dependent ribosomal degradation pathway in mammals is also distinct from previously reported ribophagy.

As described above, new ribosome synthesis and the initiation of protein synthesis are inhibited when cells are starved. However, some ribosomes synthesize the proteins required for adaptation to stressful conditions. Therefore, random engulfment of cytoplasmic ribosomes in autophagosomes appears to be inefficient. Because NUFIP1 and Rsa1p are involved in ribosome biosynthesis in the nucleus, it is possible that the ribosomes targeted for selective degradation are not those in the cytoplasm, but those in the composition process in the nucleus. Under stressful conditions, incomplete ribosomes in the nucleus are unnecessary; therefore, they are likely targets of selective degradation. Accordingly, the selective degradation of ribosomes shown here does not interfere with ribosomes attempting to translate stress-response factors into the cytoplasm. Alternatively, after translocation from the nucleus to the cytoplasm, Rsa1p may selectively bind to ribosomes that translate specific mRNAs, such as those encoding amino acid biosynthesis factors and ribosomal proteins, and deliver them to autophagosomes (44). Our structural analysis suggested that the ribosomal protein eL8 interacts with Rsa1p (Fig. 4*D*). As previously mentioned, *S. cerevisiae* has two paralogs of eL8: eL8A and eL8B. Although there are few studies on these paralogs, eL8A and eL8B have been reported to be functionally interchangeable, and changes in carbon sources alter their relative proportions within ribosomes (45). However, the availability of a nitrogen source does not influence the interaction between ribosomes and Rsa1p, as Rsa1p binds to ribosomes prepared from both conditions almost comparable (Fig. 4*C*). In addition, the structural basis for the interaction between Rsa1p and Atg8p should also be investigated. Recently, Ibrahim *et al.* predicted proteins containing Atg8p-interacting motif (AIM) and Atg8p using AlphaFold2 (46). The AIM, also known as the LC3-interacting region, is represented by [W/F/Y]xx[L/I/V] (47), and two potential AIMs, ^81^FHPI and ^144^YEDV, are identified in Rsa1p. We predicted the complex structures of Rsa1p and Atg8p using AlphaFold2 (Fig. S5) (37, 48). When the structure of Rsa1p alone was predicted, most regions were disordered, and the total predicted aligned error (PAE) was relatively low (Fig. S5*A*, left panel). However, PAE of residues within ^144^YEDV, one of the candidates for the Atg8p-interacting motif in Rsa1p, significantly improved in the predicted structure of Rsa1p complexed with Atg8p (Fig. S5*A*, right panel). Moreover, in the predicted complex structure (Fig. S5*B*), the ^144^YEDV residues (shown in blue lines) were located near the cavity of Atg8p (shown in gray surface), suggesting that ^144^YEDV is a binding site for Atg8p.

GFP cleavage and ALP assays revealed that the *rny1*Δ strain reduces bulk autophagy activity (Fig. 5), indicating that RNA accumulation in vacuoles harms cells. Although other additional cause(s) cannot be denied, the accumulated RNA inhibits the adaptation of the *rny1*Δ strain to stressed conditions. In addition to stress responses, in *Arabidopsis thaliana*, the RNase T2 null mutant accumulates RNA and ribosomes in autophagosomes and autophagic bodies even under nutrient-rich conditions, suggesting the maintenance of homeostasis (34). RNA accumulation has also been observed in the lysosomes of neurons in RNase T2-deficient zebrafish (5). The RNase T2 null zebrafish shows an abnormal MRI pattern in the brain, which is assumed to reproduce the cystic leukoencephalopathy observed in humans (9); which also indicates that the accumulation of RNA in lysosomes is detrimental.

After glycosylation, Rny1p is usually secreted from the cell and anchored to the host cell wall (Fig. 7) (20). Our results indicated that the C-terminal extension is required for the interaction, and possibly, the sugar chain of the cell wall serves as a scaffold (Fig. 6, *B*–*D*). However, the mechanism by which Rny1p is differentially transported into the vacuole and extracellular space remains unclear. After nitrogen starvation was induced, the amount of extracellular Rny1p decreased, suggesting that starvation changed the cellular trafficking of Rny1p (Fig. 6*E*). The physiological role of cell wall anchoring remains also unknown. Some extracellular RNAs are thought to bind to cell membranes to regulate ion permeability (49), and Rny1p degrades these RNAs for regulation (19). Our studies have shown that the C-terminal extension is required for growth, at least at high temperatures (Fig. S6). Further studies are necessary to clarify the function of the C-terminal extension of Rny1p. Some RNase T2 proteins, including Rny1p, possess the C-terminal extensions. Fungal RNase T2 proteins, including Irp3, are suggested to share the structure and function. To obtain the insight into the conservation of the C-terminal extension among RNase T2 family members, we performed phylogenetic analysis using the Graph Splitting method (50). As a result, fungal RNase T2 diverged from other RNase T2 proteins early in the phylogenetic tree, suggesting that they evolved independently (Fig. S4*A*). Nevertheless, the X-ray crystal structures of plant *Pyrus pyrifolia* and *Homo sapiens* RNase T2 were well superimposed on the predicted structure of the RNase domain of Rny1p (Fig. S7, *B* and *C*). The presence of RNase T2 in almost all organisms suggests that cells retained RNase T2 at an early stage of evolution. Over its long evolutionary history, RNase T2 has played a specific role in many species. Acquisition of the C-terminal extension may be one reason for the diverse roles of enzymatically simple ribonucleases in different species. To understand this process better, the structure and function of the C-terminal extension of the RNase T2 family must be studied.

## Experimental procedures

### Yeast strains, plasmids, and culture conditions

The yeast strains and plasmids used in this study are listed in Tables S1 and S2. The pRS316[GFP-ATG8] plasmid, expressing Atg8p fused with GFP at the N-terminus, was provided by the National Bio-Resource Project (NBRP) – Yeast, Japan. The strain chromosomally expressing Rpl25p-GFP (30) was a gift from Professor Hiroshi Takagi. Plasmids pGMH10, pGMH20, and pGMU10 were provided by RIKEN BRC through the National Bio-Resource Project of the MEXT, Japan (cat. RDB01955, RDB01956, and RDB01961, respectively). The strains were cultured in YPD (1% yeast extract, 2% polypeptone, and 2% glucose) or synthetic dextrose (SD) medium (0.67% nitrogen base without amino acids and 2% glucose) at 30 °C. For gene expression under the control of the *GAL1* promoter, cells were pre-cultivated overnight in a synthetic medium containing 2% raffinose instead of glucose (SRaf medium). After washing, the cells were inoculated into synthetic galactose (SG) medium to achieve an optical density at 660 nm (OD_660_) of 0.1, and cultivation was reinitiated. Rapamycin (100 nM) was used at the mid-log phase (OD_660_=0.3–0.4) to induce cell starvation. Nitrogen-depleted (SD (-N)) medium containing 0.17% yeast nitrogen base without amino acids, ammonium sulfate, or that containing 2% glucose instead of glucose was used to induce nitrogen starvation.

### Reagents

Rapamycin (a mixture of isomers) was purchased from FUJIFILM Wako Pure Chemical Corporation and dissolved in DMSO. LongLife Zymolyase and endoglycosidase H (Endo H) were purchased from G-Bioscience and New England Biolabs, respectively. PMSF was purchased from Nacalai tesque and dissolved in isopropyl alcohol. Protease Inhibitor Cocktail was purchased from Sigma-Aldrich.

### Plasmid construction

Genes for wild-type Rny1p and lacking the C-terminal extension (Rny1pΔC) without stop codons were PCR amplified, introducing EcoRI and XhoI sites at the 5′ and 3′ ends, respectively. Subsequently, they were digested with EcoRI and XhoI and inserted into the same restriction sites of pYN735 to introduce a 3xFLAG tag gene, resulting in fusion genes with a 3xFLAG tag coding region at the 3′ ends. These genes were then excised with EcoRI and SalI and inserted into the same restriction sites of pGMH20, a high-copy number plasmid containing the *GAL1* promoter and *HIS3* marker. The resulting plasmids, pGMH20-*RNY1*-*F* and pGMH20-*RNY1*ΔC*-F*, express Rny1p and its variant lacking the C-terminal extension, respectively, each with a 3xFLAG-tag fused at their C-termini. A plasmid expressing catalytically inactive Rny1p was prepared by introducing mutations to exchange the 87th and 160th histidine codons with phenylalanine, using the QuickChange site-directed mutagenesis method. The resulting plasmid was designated pGMH20-*RNY1*-ci-*F* (ci: Catalytically Inactive). These plasmids were then introduced into the BY4742 strain, and the genes were expressed under the control of the *GAL1* promoter. Genes for mCherry-fused Rsa1p and GFP-fused Rsa1p were PCR-amplified, introducing SmaI and SalI sites at the 5′ and 3′ ends, respectively. Subsequently, they were digested with SmaI and SalI and inserted into the same restriction sites of pGMU10, a low-copy number plasmid containing the *GAL1* promoter and *URA3* marker. The resulting plasmids named pGMU10-*mCherry-RSA1* or pGMU10-*GFP-RSA1* was introduced into the strain chromosomally expressing Rpl25p-GFP or the *rsa1*Δ strain. A plasmid co-expressing Rsa1p and Hit1p was constructed as follows: First, the DNA fragment of *HIT1* was PCR-amplified and cloned into multi-cloning site two of pETDuet-1 using the in-fusion HD Cloning Kit (Takara Bio). Second, the DNA fragment of *RSA1* was amplified by PCR to introduce XbaI and SalI sites at the 5′ and 3′ ends, respectively. Finally, it was digested with XbaI and SalI, inserted into the same restriction sites of the multi-cloning site one of pETDuet-1; the resultant plasmid was designated as pETDuet1-*RSA1*-*HIT1*. A plasmid expressing FLAG-Atg8p named pET24d-*F-ATG8* was constructed as follows: An *ATG8*-containing fragment was obtained by digesting pRS316[GFP-ATG8] with NcoI and SalI and transferred into the pET24d(+) vector. Then, a FLAG tag-coding gene was introduced at the 5′ end of *ATG8* using the in-fusion kit.

### Strain construction

Genes for wild-type *RNY1* and *HIS3* cassette were PCR amplified using pGMH20-*RNY1-F* as a template to introduce homologous regions at the 5′ and 3′ sides of the genomic *RNY1*. The amplified fragment was transduced into the BY4742 strain and the genomic *RNY1* gene was replaced by double-crossover homologous recombination. Transformants were plated on SD medium containing leucine, lysine, and uracil at 30 °C and incubated until visible colonies were obtained. Transformants in which the native *RNY1* gene was successfully replaced by the transduced gene were screened using colony PCR. Similarly, DNA fragments containing genes encoding Rny1pΔC-F, Rny1p-F-ci, and the *HIS3* cassette were also amplified from pGMH20-*RNY1*ΔC*-F*, pGMH20-*RNY1*-*F*-ci, and empty pGMH20 as described above and integrated into the genome. The *RNY1* gene of these transformants was amplified by PCR and sequenced to verify proper integration of the transduced fragments. The resultant transformants chromosomally expressing Rny1p-F, Rny1pΔC-F, and Rny1pΔC-F-ci were designated as *rny1*Δ+WT, *rny1*Δ+ΔC, and *rny1*Δ+ci, respectively. The *rny1*Δ+His3, which was constructed by replacing *RNY1* with *HIS3* cassette, was used as a control.

### Measurement of cell growth

Cells were grown in YPD medium at 30 °C until OD_660_ reached 0.3 to 0.4. These cultures were serially diluted five-fold, spotted on plates containing rapamycin (5 nM), and cultivated at 30 °C until visible colonies formed. To assess the effect of starvation on cell growth over time, the wild-type and *rny1*Δ strains were grown in a liquid YPD medium until the OD_660_ reached 0.4, and then rapamycin (100 nM) was added. OD was automatically monitored using an OD-MonitorC&T instrument (TAITEC). In all experiments involving rapamycin treatment, DMSO was used as the solvent for rapamycin and was added as a mock treatment, confirming that the concentration of DMSO used did not affect the growth of the wild-type strains. To examine the extent of RNA degradation in cells cultured in YPD, the OD_660_ was measured every hour, and the cell cultures were diluted with YPD to maintain an OD_660_ between 0.85 and 1.3 (2). Since the *CDC48* gene is essential, the CDC48 Tet-off strain purchased from the Yeast Tet-Promoters Hughes Collection (Open Biosystems) was used; the addition of DOX suppressed the expression of *CDC48*. CDC48 Tet-Off cells at an OD_660_ of 0.1 were cultivated with or without 10 μg/ml of DOX in a YPD medium. After 14 h of cultivation, rapamycin (100 nM) was added, and the culture was continued for 2 h. The cells were subsequently sampled, RNA and vacuoles were stained, and fluorescence microscopic analysis was performed.

### Quantification of gel signal intensity

The signal intensity of the gel was quantified as reported in the literature (51).

### RNA preparation

Total RNA was extracted using the hot phenol method. The collected cells were resuspended in 400 μl of AE buffer (50 mM sodium acetate (pH 5.0) and 10 mM EDTA) and 40 μl of 10% SDS, and briefly mixed at room temperature. Further, 500 μl of AE-buffer-equilibrated phenol (pre-warmed at 65 °C) was immediately added, and the samples were vigorously mixed for 1 min. Samples were incubated at 65 °C for 5 min and mixed for 5 s every 30 s. The samples were centrifugated, and 300 μl of the resulting supernatant was collected. Subsequently, 400 μl of phenol: chloroform (1:1) and 100 μl of water were added, and the samples were vigorously mixed for 1 min. Three hundred microliters of the supernatant was collected, and 400 μl of chloroform: isoamyl alcohol (24:1) and 100 μl of water were added, and the samples were vigorously mixed for 1 min. After centrifugation, 300 μl of the resulting supernatant was collected, and total RNA was recovered by ethanol precipitation. For electrophoresis, the volume of the rRNA solution to be loaded onto a denaturing agarose gel was normalized according to cell number (estimated by the OD_660_ value) to compare the amount of rRNA per cell. Using Coulter counters (Multisizer 3, Beckman Coulter), we confirmed that there was no significant difference in the cell sizes of the wild type and mutant strains under starvation conditions. After electrophoresis, the gel was stained with SYBR Gold (Thermo Fisher Scientific) to visualize the RNA.

### Quantification of RNA by RT-qPCR

An equivalent volume of culture medium was sampled from each culture test tube. Total RNA was prepared using the hot-phenol method and purified using the NucleoSpin RNA Clean-up Kit (Takara Bio). Subsequently, 100 ng of each total RNA sample was reverse-transcribed using the PrimeScript RT Master Mix (Perfect Real Time) (Takara Bio). qPCR was performed using the QuantiTect SYBR Green PCR Kit (QIAGEN). A calibration curve was constructed to calculate the quantity of each rRNA contained in 100 ng of total RNA. Based on this, the absolute amounts of 35S, 18S, and 25S rRNA in the culture medium per unit volume sampled were calculated. *ACT1* mRNA was used as an internal control. The quantified rRNA was normalized to the number of cells per unit volume (estimated using the OD_660_ value, as aforementioned). The primer sequences are listed in Table S3.

### Western blotting

Whole proteins were prepared as described previously method (52). To detect GFP-Rsa1p, GFP-Atg8p and Rpl25p-GFP, an anti-GFP polyclonal antibody (Medical & Biological Laboratories) and rabbit horseradish peroxidase-conjugated IgG whole antibody (Sigma-Aldrich) were used as primary and secondary antibodies, respectively. An anti-DYKDDDDK tag, monoclonal antibody (FUJIFILM Wako Pure Chemical Corporation), anti-mouse IgG, and sheep horseradish peroxidase-linked whole antibody (GE Healthcare) were used to detect N-terminally FLAG-tagged Atg8p and C-terminally FLAG-tagged Rny1p. To detect His-tagged Rsa1p, an anti 6x histidine MoAb (9C11) (FUJIFILM Wako Pure Chemical Corporation) was used. The anti-Rpl3p monoclonal antibody was prepared using a hybridoma purchased from the Developmental Studies Hybridoma Bank.

### ALP assay

The alkaline phosphatase (ALP) activity assay was performed as described previously (36). Strain PhoΔ60 was constructed based on the BY4742 background. The protein concentrations were determined using the Bradford method. α-Naphthyl phosphate disodium salt (Sigma-Aldrich) was used as a substrate, and fluorescence was measured at emission and excitation wavelengths of 360 nm and 465 nm, respectively.

### *In vitro* binding assay

*E. coli* Rosetta 2 (DE3) was transformed with the plasmid pETDuet1-*RSA1*-*HIT1* and then cultivated in 100 ml LB medium at 37 °C. When the OD_660_ reached 0.4, β-D-thiogalactopyranoside (IPTG) was added to the culture at a final concentration of 1 mM to induce gene expression, and the culture was further incubated for 3 h at 37 °C. The cells were harvested by centrifugation and the cell pellet was resuspended in 15 ml of lysis/wash buffer A (20 mM Tris-HCl (pH 7.5), 500 mM NaCl, and 20 mM imidazole (pH 8.0)), followed by sonication on ice to lyse the cells. Cell debris was removed by centrifugation at 17,000 rpm for 20 min. The supernatant was applied to the Ni-NTA column and the resin was washed with 20 ml of lysis/wash buffer A. His-tagged Rsa1p complexed with Hit1p was eluted in 3 ml of elution buffer (20 mM Tris-HCl (pH 7.5), 500 mM NaCl, and 250 mM imidazole (pH 8.0)). The purified protein sample was concentrated using a Vivaspin 20 device (10,000 MWCO; Sartorius) and stored in a buffer containing 50 mM Na_3_PO_4_, 300 mM NaCl, and 0.01% Tween-20. The plasmid pET24d-*F-ATG8* was introduced to the *E. coli* Rosetta 2 (DE3) and cultivated as described above. The cell pellet was resuspended in 15 ml of lysis buffer B (20 mM Tris-HCl (pH 7.5) and 500 mM NaCl), followed by sonication on ice to lyse the cells. Cell debris was removed as described above and the lysate containing FLAG-tagged Atg8p was obtained. A pull-down assay was conducted using Dynabeads His-Tag Isolation and Pull-down kit (Invitrogen). 100 μl of the purified His-tagged Rsa1p and 700 μl of the lysate containing FLAG-tagged Atg8p were added to 2 mg of the magnetic beads. The mixture was then incubated overnight with stirring in a rotator at 4 °C, followed by washing the beads 4 times with wash buffer (50 mM Na_3_PO_4_, 300 mM NaCl, and 0.1% Triton X-100). Subsequently, Rsa1p and interacting proteins were eluted with a buffer containing 50 mM Na_3_PO_4_, 300 mM NaCl, 300 mM imidazole (pH 8.0), and 0.1% Triton X-100, followed by SDS-PAGE and western blotting using anti-His-tag and anti-FLAG-tag antibodies.

Yeast ribosomes were extracted from the *atg2*Δ strain chromosomally expressing Rpl25p-GFP. Cells were grown in SD medium at 30 °C. When the OD_660_ reached 0.4, a cell culture was collected and suspended in 10 ml of SD (-N) medium and further cultivated for 1 h. These cell cultures were collected, suspended in 500 μl of crushing buffer (10 mM Tris-HCl (pH 7.5), 10 mM MgCl_2_, and 60 mM NH_4_Cl), and disrupted with glass beads using a Multi-beads shocker (Yasui Kikai). The cell debris were removed by centrifugation, and ribosomes were prepared from cell lysates by ultrafiltration using Nanosep OMEGA 300k (Pall corporation). A ribosome-binding assay was performed as follows: ribosomes and His-tagged Rsa1p were mixed in 100 μl of association buffer (20 mM HEPES-KOH (pH 7.9), 150 mM NaCl, 10 mM MgCl_2_, and 0.1% Triton X-100). Reaction mixtures were incubated for 30 min at 25 °C, and ribosome-containing fractions were obtained by ultrafiltration using Nanosep as described above, followed by SDS-PAGE and Western blotting using anti-His-tag and anti-GFP-tag antibodies.

### Endo H treatment, concentration of cell culture medium, and preparation of spheroplasts

After 3 h of nitrogen starvation, cells were collected and suspended in 100 μl of SD (-N) medium and 1 μl of 2-Mercaptoethanol, vortexed for 1 min, and then placed on ice. Further, 15 units of LongLife Zymolyase were added, and the mixture was incubated for 1 h at 37 °C. The spheroplast and cell wall fractions were separated by centrifugation. To remove glycosylation, 500 units of Endo H were added and incubated for 1 h at 37 °C. The cell culture concentration was determined using a Vivaspin Turbo 15 device (30,000 MWCO; Sartorius).

### PMSF treatment and formation of the autophagic body for the detection of Rsa1p-ribosome interaction

PMSF at a final concentration of 1 mM was added to the medium when nitrogen starvation was induced. For the fluorescence microscopy observation, cells were cultured overnight, and the cells were collected and fixed in 1 ml of PBS containing 10% formaldehyde. For the ribosome fractionation, cells were cultured 3 h after the induction of nitrogen starvation, and these cell cultures were collected and suspended in the buffer (20 mM HEPES-KOH (pH 7.4), 2.5 mM MgCl_2_, 150 mM NaCl, 0.5% protease inhibitor cocktail, and 1 mM PMSF). Cell disruption and ribosome fractionation were performed as described above.

### Fluorescence microscopic analysis

FM-4-64 Dye (Thermo Fisher Scientific), Hoechst 33342 (Nacalai tesque), and GRG post stain 10,000 × in water (Biocraft) were used to stain the vacuole, nucleus, and the RNA accumulated in the vacuole, respectively. An Olympus BX40 and BX53 microscope with a fluorescence system (Olympus) and an FV1200 laser scanning microscope (Olympus) were used for imaging.

### Statistical analysis

Statistical analyses were performed using the JASP software (53). Statistical significance between groups was analyzed by Student's *t* test for comparisons between two groups or one-way ANOVA and Tukey-Kramer *post hoc* test for comparisons among three or more groups. Results were considered statistically significant at *p* < 0.05.

### AlphaFold2 modeling and structural alignments

Structural models were predicted using the AlphaFold2 Colab web server (https://colab.research.google.com/github/sokrypton/ColabFold/blob/main/AlphaFold2.ipynb) (37, 54) or its local version (https://github.com/YoshitakaMo/localcolabfold) (37, 54). The reliability of the model was estimated using the predicted local distance difference test (pLDDT) and PAE matrices. Structural alignments were performed and figures were constructed using PyMOL 2.5.0 (Schrodinger). The pLDDT score was mapped onto the structure using the PSICO plugin (https://github.com/YoshitakaMo/pymol-psico).

### Phylogenetic analysis

Fifty-five amino acid sequences of proteins belonging to the RNase T2 superfamily listed in Swiss-Prot were obtained using UniProt. Homologous amino acid sequences were removed by CD-HIT (55). The amino acid sequence of *Irpex lacteux* RNase T2 (Irp3, GenBank ID: BAC00516.1) was added. A phylogenetic tree was constructed using the Graph Splitting (50).

## Data availability

The original contributions presented in this study are included in the article/supporting information. Further inquiries can be directed at the corresponding author.

## Supporting information

This article contains supporting information (30).

## Conflict of interest

The authors declare that they have no conflicts of interest with the contents of this article.
