# Supplementary material for: The ribonuclease RNase T2 mediates selective autophagy of ribosomes induced by starvation in Saccharomyces cerevisiae
Source: J Biol Chem. 2025 Apr 26;301(6):108554. doi: 10.1016/j.jbc.2025.108554 (PMC12152620; doi:10.1016/j.jbc.2025.108554)
Supplement: Supporting information [file mmc1.docx]

**Supporting Information**

**Title**

The ribonuclease RNase T2 mediates selective autophagy of ribosomes induced by starvation in *Saccharomyces cerevisiae*

**Author Affiliations**

Atsushi Minami^1,‡^, Kohei Nishi^1,‡^, Rikusui Yamada^1,‡^, Gai Jinnai^1^, Hikari Shima^1^, Sakiko Oishi^1^, Hirofumi Akagawa^1^, Toshihiro Aono^2^, Makoto Hidaka^1^, Haruhiko Masaki^1^, Tomohisa Kuzuyama^1,3^, Yoichi Noda^1,3^, and Tetsuhiro Ogawa^1,3,*^

^1^ Department of Biotechnology, The University of Tokyo, Yayoi, Bunkyo-ku, Tokyo, Japan

^2^ Agro-Biotechnology Research Center (AgTECH), The University of Tokyo, Yayoi, Bunkyo-ku, Tokyo, Japan

^3^ Collaborative Research Institute for Innovative Microbiology (CRIIM), The University of Tokyo, Yayoi, Bunkyo-ku, Tokyo, Japan

‡ These authors contributed equally to this work

***** For correspondence: Tetsuhiro Ogawa, [atetsu@g.ecc.u-tokyo.ac.jp](mailto:atetsu@g.ecc.u-tokyo.ac.jp).

Present address for Toshihiro Aono: National Agriculture and Food Research Organization, Tsukuba‐city, Ibaraki, Japan.

**Running Title**

Mechanism of the stress response by yeast RNase T2

**Figure S1**

**Figure S1. Rny1p degrades RNA in the vacuole upon rapamycin treatment, as well as nitrogen starvation.** (A) After rapamycin treatment, the degradation of rRNAs in wild-type and *rny1*Δ strains at each time point was observed by electrophoresis using a denaturing agarose gel. The ratio of rRNA remaining after 3 h of rapamycin treatment in wild-type and *rny1*Δ strains was calculated as follows: rRNA (%) = 100 × (the band intensity of rRNA prepared from cells cultivated with 3 h of rapamycin treatment)/(the band intensity of rRNA prepared from cells cultivated without 3 h of rapamycin treatment). The arrowhead indicates small RNAs containing tRNAs. 18S and 25S rRNA were more abundant in the *rny1*Δ strain compared to the wild-type strain after rapamycin treatment. (B) The wild-type and *rny1*Δ strains in the mid-log phase were further cultivated with or without rapamycin (100 nM) for 2 h. “Rap” in panels means the addition of rapamycin. Cultivation, rapamycin treatment, cell staining, and fluorescent microscopic analysis were performed as in Figure 2. RNA accumulation was observed in the vacuole of the *rny1*Δ strain by the addition of rapamycin. The scale bar represents 10 µm.

**Figure S2**

**Figure S2. Bulk autophagic activity is not perturbed by Rsa1p-dependent selective autophagy of ribosomes.** (A) Nitrogen starvation was induced in wild-type, *rsa1*Δ, *rny1*Δ, and *atg2*Δ strains, and then the cells were collected at the indicated time points. Cell lysate preparation and Western blotting were performed as shown in Figures 3A and 5A. GFP-Atg8p and GFP fragments are indicated by closed and opened arrowheads, respectively. The percentage of the GFP cleavage was calculated as follows: cleavage (%) = 100 × (intensity of band from GFP fragment)/(intensity of band from GFP-Atg8p + intensity of band from GFP fragment). No significant differences in the appearance of GFP fragments over time were observed in both wild-type and *rsa1*∆ strains. (B) Nitrogen starvation was induced in wild-type, *rsa1*Δ, *rny1*Δ, and *atg2*Δ strains expressing Pho8Δ60p for 6 h, and then ALP assay was performed as shown Figure 5B. Data are presented as means ± standard error from 3 independent experiments. Multiple comparison test was conducted at each point in time. Different letters indicate significant differences (P < 0.05, Tukey-Kramer test). The bulk autophagic activity of the *rsa1*∆ strain is almost the same as that of the wild-type strain, indicating that selective autophagy of ribosomes does not interfere with bulk autophagy.

**Figure S3**

**Figure S3. Rny1pΔC-F is glycosylated and released into the medium.** Wild-type strain harboring pGMH20 (an empty vector), pGMH20-*RNY1*-*F*, or pGMH20-*RNY1*ΔC-*F* (leveled as ctrl, WT, or ΔC, respectively) was cultivated in an SG medium as shown in Figure 6D. Then, cell lysate and concentrated medium were prepared, and Western blotting was performed using an anti-FLAG antibody. The asterisk indicates glycosylated Rny1pΔC-F observed in the concentrated medium. “C” and “M” labeled above the panel indicate that the cell lysate or concentrated medium was loaded, respectively.

**Figure S4**

**Figure S4 (continued)**

**Figure S4. Diversity of the C-terminal extensions of RNase T2.** (A) A phylogenetic tree of the RNase T2 family was created based on amino acid sequences. RNase T2 of classical swine fever (CSF) virus, Arabidopsis thaliana, Caenorhabditis elegans, Irpex lacteus, and S. cerevisiae are discussed in this paper, and their genus names are colored in red. Each bar drawn to the right of each organism’s name represents the protein sequence length, with the RNase T2 domain (IPR001568) highlighted in blue. The right edges of the blue bars, representing the domain end positions, are aligned for comparison. (B) The structure of *Irpex lacteux* RNase T2 (Irp3) was predicted by AlphaFold2, with confidence levels mapped onto the structure. (C) Structural alignment of the predicted structures of the C-terminal extension of Rny1p (green) and Irp3 (magenta). (D) (upper panel) Structural alignment of the predicted structures of the fungal RNase T2 C-terminal extension. Salmon: *Emericella nidulans*; green: *Neosartorya fumigata*; yellow: *Yarrowia lipolytica*; slate: *Debaryomyces hansenii*; cyan: *Candida albicans* RNY1B; magenta: *Candida albicans* RNY1A; orange: *Kluyveromyces lactis*; grey: *Candida glabrata*; deep teal: *Saccharomyces cerevisiae*. (lower panel) RMSD values of these structural alignments. (E) The structure of E^rns^, a CSF virus RNase T2, was predicted using AlphaFold2, with confidence levels mapped onto the structure.

**Figure S5**

**Figure S5. The structural prediction of Rsa1p and Atg8p by AlphaFold2.** (A) The PAE matrixes of the AlphaFold2 prediction of Rsa1p in isolation (left panel) and the Rsa1p-Atg8p complex (right panel). The yellow arrowhead indicates the putative Atg8p-binding residues in Rsa1p. (B) The predicted complex structure of Rsa1p (depicted as a ribbon model with confidence levels mapped onto the structure) and Atg8p (shown as a surface model).

**Figure S6**

**Figure S6. The C-terminal extension is required for growth at high temperature.** *rny1*Δ strains carrying the plasmid pMMH20, which was constructed by replacing the *GAL1* promoter with *MET3* promoter, encoding Rny1p-F, Rny1pΔC-F, Rny1pΔN-F (Rny1p-F lacking the N-terminal signal peptide), or Rny1p-F-ci were cultured in SD medium to log phase. The *MET3* promoter is immediately activated by the depletion of methionine in the medium. Then, they were serially diluted (1:3) and spotted onto two solid SG medium plates. The plates were incubated at 30 or 37 °C.

**Figure S7**

**Figure S7. Structural conservation of the RNase domain of eukaryotic RNase T2.** (A) MSA of representative RNase T2 sequences in eukaryotes. The sequences were aligned using ClustalW (https://www.genome.jp/tools-bin/clustalw). The labels shown on the left of the amino acid sequences are as follows: S_cerevisiae: RNase domain of Rny1p from *S. cerevisiae* predicted by AlphaFold2, H_sapiens: Ribonuclease T2 from *Homo sapiens* (PDB ID: 3T0O), and P_pyrifolia: Ribonuclease S-3 from *Pyrus pyrifolia* (PDB ID: 1IQQ). (B) Structural alignment of the predicted RNase domain of Rny1p (labeled as Rny1p) with the crystal structures of *H. sapiens* (labeled as HsRNase T2) and *P. pyrifolia* (labeled as PpS-RNase). (C) RMSD values of (B) for structural alignments.

**Table S1. Strains used in this study.**

| **Strain** | **Genotype** | **Source** |
| --- | --- | --- |
| BY4741 | *MAT*a *his3*Δ*1* *leu2*Δ*0* *met15*Δ*0* *ura3*Δ*0* | Euroscarf |
| BY4742 | *MAT*α *his3*Δ*1* *leu2*Δ*0* *lys2*Δ*0* *ura3*Δ*0* | Euroscarf |
| BY4743 | *MAT*a/α *his3*Δ*1/his3Δ1* *leu2*Δ*0/leu2Δ0* *met15*Δ*0/MET15* *LYS2*/*lys2Δ0 ura3*Δ*0/ura3Δ0* | Euroscarf |
| Y12129 | *MAT*α *his3*Δ*1* *leu2*Δ*0* *lys2*Δ*0* *ura3*Δ*0* *rny1*Δ::*kanMX4* | Euroscarf |
| Y16148 | *MAT*α *his3*Δ*1* *leu2*Δ*0* *lys2*Δ*0* *ura3*Δ*0* *ubp3*Δ::*kanMX4* | Euroscarf |
| Y11099 | *MAT*α *his3*Δ*1* *leu2*Δ*0* *lys2*Δ*0* *ura3*Δ*0* *rsa1*Δ::*kanMX4* | Euroscarf |
| Y11970 | *MAT*α *his3*Δ*1* *leu2*Δ*0* *lys2*Δ*0* *ura3*Δ*0* *atg2*Δ::*kanMX4* | Euroscarf |
| Y15063 | *MATα his3Δ1 leu2Δ0 lys2Δ0 ura3Δ0 ufd3Δ::kanMX4* | Euroscarf |
| *ubp3*Δ*rny1*Δ | *MATα his3Δ1 leu2Δ0 lys2Δ0 ura3Δ0 ubp3Δ::kanMX4 HIS3::rny1*Δ | This study |
| *rsa1*Δ*rny1*Δ | *MAT*α *his3*Δ*1* *leu2*Δ*0* *lys2*Δ*0* *ura3*Δ*0* *rsa1*Δ::*kanMX4* *rny1*Δ::*URA3* | This study |
| *ufd3*Δ*rny1*Δ | *MATα his3Δ1 leu2Δ0 lys2Δ0 ura3Δ0 ufd3Δ::kanMX4 HIS3::rny1*Δ | This study |
| CDC48 tet-off | *MATa his3-1 leu2-Δ0 ura3-Δ0 URA3::CMV-tTA, KanMX::tetO_7_CYC_TATA_CDC48* | Hughes Collection |
| CDC48 tet-off *rny1*Δ | *MATa his3-1 leu2-Δ0 ura3-Δ0 URA3::CMV-tTA, KanMX::tetO_7_CYC_TATA_CDC48 HIS3::rny1*Δ | This study |
| pho8Δ60 | *MAT*α *his3*Δ*1* *leu2*Δ*0* *lys2*Δ*0* *ura3*Δ*0* *pho8*Δ*60*::*clonNAT* | This study |
| pho8Δ60*rny1*Δ | *MAT*α *his3*Δ*1* *leu2*Δ*0* *lys2*Δ*0* *ura3*Δ*0* *pho8*Δ*60*::*clonNAT* *rny1*Δ::*kanMX4* | This study |
| pho8Δ60*rsa1*Δ | *MAT*α *his3*Δ*1* *leu2*Δ*0* *lys2*Δ*0* *ura3*Δ*0* *pho8*Δ*60*::*clonNAT* *rsa1*Δ::*kanMX4* | This study |
| pho8Δ60*atg2*Δ | *MAT*α *his3*Δ*1* *leu2*Δ*0* *lys2*Δ*0* *ura3*Δ*0* *pho8*Δ*60*::*clonNAT* *atg2*Δ::*kanMX4* | This study |
| *rny1*Δ+His3 | *MAT*α *his3*Δ*1* *leu2*Δ*0* *lys2*Δ*0* *ura3*Δ*0* *rny1*Δ::*HIS3* | This study |
| *rny1*Δ+WT | *MAT*α *his3*Δ*1* *leu2*Δ*0* *lys2*Δ*0* *ura3*Δ*0* *rny1*Δ::*RNY1-F*::*HIS3* | This study |
| *rny1*Δ+ΔC | *MAT*α *his3*Δ*1* *leu2*Δ*0* *lys2*Δ*0* *ura3*Δ*0* *rny1*Δ::*RNY1*Δ*C-F*::*HIS3* | This study |
| *rny1*Δ+ci | *MAT*α *his3*Δ*1* *leu2*Δ*0* *lys2*Δ*0* *ura3*Δ*0* *rny1*Δ::*RNY1-F-*ci::*HIS3* | This study |
| RPL25-GFP | *MAT*a *his*3Δ*1* *leu2*Δ*0* *met15*Δ*0* *ura3*Δ0 *rpl25*Δ::*RPL25-GFP-hph* | ref. 30 |
| RPL25-GFP*rsa1*Δ | *MAT*a *his3*Δ*1* *leu2*Δ*0* *met15*Δ*0* *ura3*Δ*0* *rpl25*Δ::*RPL25-GFP-hph* *rsa1*Δ::*kanMX4* | This study |
| RPL25-GFP*atg2*Δ | *MAT*a *his3*Δ*1* *leu2*Δ*0* *met15*Δ*0* *ura3*Δ*0* *rpl25*Δ::*RPL25-GFP-hph* *atg2*Δ::*kanMX4* | This study |

**Table S2. Plasmids used in this study.**

| **Name** | **Description** | **Source** |
| --- | --- | --- |
| pRS316[GFP-ATG8] | expressing GFP-Atg8p | NBRP, Japan |
| pGMH20 | a high-copy plasmid coding *GAL1* promoter | RIKEN, BRC |
| pGMH20-*RNY1-F* | expressing FLAG-tagged Rny1p | This study |
| pGMH20-*RNY1*ΔC-*F* | expressing FLAG-tagged Rny1p lacking the C-terminal extension | This study |
| pGMH20-*RNY1(H87F/H160F)-F* | expressing FLAG-tagged Rny1p (H87F/H160F) mutant | This study |
| pGMU10 | a low-copy plasmid coding *GAL1* promoter | RIKEN, BRC |
| pGMU10-*mCherry-RSA1* | expressing mCherry-Rsa1p under *GAL1* promoter | This study |
| pGMU10-*GFP-RSA1* | expressing GFP-Rsa1p under *GAL1* promoter | This study |
| pMMH20 | a high-copy plasmid whose *GAL1* promoter is replaced with *MET3* promoter | This study |
| pMMH20-*RNY1-F* | expressing FLAG-tagged Rny1p under *MET3* promoter | This study |
| pMMH20-*RNY1*ΔN-*F* | expressing FLAG-tagged Rny1p lacking the N-terminal signal peptide under *MET3* promoter | This study |
| pMMH20-*RNY1*ΔC-*F* | expressing FLAG-tagged Rny1p lacking the C-terminal extension under *MET3* promoter | This study |
| pMMH20-*RNY1-F*-ci | expressing FLAG-tagged Rny1p (H87F/H160F) mutant under *MET3* promoter | This study |
| pETDuet1-*RSA1-HIT1* | co-expressing Rsa1p and Hit1p in *E. coli* | This study |
| pET24d(+)-*F-ATG8* | expressing FLAG-tagged Atg8p in PURE*frex* 1.0 | This study |

**Table S3. Primer sequences used in this study.**

| **Name** | **Forward primer(5’-3’)** | **Reverse primer(5’-3’)** |
| --- | --- | --- |
| 35S_ITS_RT | CTGGCCTTTTCATTGGATGT | TAGCCGCAGTTGGTAAAACC |
| 18S_RT | CATGGCCGTTCTTAGTTGGT | ATTGCCTCAAACTTCCATCG |
| 25S_RT | GGCTCTAAGGGTCGGGTAGT | TCTACAACAAGGCACGCAAG |
| 35S_ITS_CC | TTTTTCTTTGGGCATTCGAG | TTCGCCTAGACGCTCTCTTC |
| 18S_CC | CCTGCGGCTTAATTTGACTC | GTACAAAGGGCAGGGACGTA |
| 25S_CC | AGTTGGACGTGGGTTAGTCG | TCTACAACAAGGCACGCAAG |

**Table S4. Top 5 hits for protein structures that are structurally similar to the C-terminal extension of Rny1p by Dali search.**

| **Rank** | **PDB ID** | **Z score** | **RMSD [Å]** | **Description** |
| --- | --- | --- | --- | --- |
| 1 | 5B2H_A | 11.0 | 3.1 | HA-33 |
| 2 | 3AH2_B | 10.8 | 3.3 | Main Hemagglutinin Component |
| 3 | 2VSE_A | 10.7 | 3.2 | Mosquitocidal Toxin |
| 4 | 4IY8_A | 10.5 | 3.0 | 30K Protein 1 |
| 5 | 4LO0_A | 10.5 | 3.3 | HA-33 |
